# Sex‐Specific Neurogenic and Cognitive Responses in a Murine Model of Accelerated Aging

**DOI:** 10.1111/acel.70663

**Published:** 2026-10-09

**Authors:** Ricardo Gómez‐Oliva, Andrea Chamorro‐Francisco, Isabel Atienza‐Navarro, Livia Carrascal, María D. Freire‐Aragón, Rosario Hernández‐Galán, Pedro Nunez‐Abades, Mónica García‐Alloza, Carmen Castro

**Affiliations:** ^1^ Área de Fisiología, Facultad de Medicina Universidad de Cádiz Cádiz Spain; ^2^ Instituto de Investigación e Innovación Biomédica de Cádiz Cádiz Spain; ^3^ Departamento de Fisiología, Facultad de Farmacia Universidad de Sevilla Sevilla Spain; ^4^ Hospital Universitario Puerta del Mar de Cádiz Cádiz Spain; ^5^ Departamento de Química Orgánica, Facultad de Ciencias Universidad de Cádiz Cádiz Spain

**Keywords:** aging, cognitive performance, neurogenesis, sex‐differences, therapy

## Abstract

Aging is associated with cognitive deterioration accompanied by a reduction in hippocampal neurogenesis. Murine models have been widely used to study aging and age‐related cognitive decline, as they recapitulate many of the key features of the degenerative process. Among these, the SAMP8 strain represents a well‐established model of accelerated aging, characterized by early‐onset and progressive cognitive impairment, Alzheimer's disease‐like neuropathology, and an initial increase in hippocampal neurogenesis that ultimately depletes the neural stem cell pool. Notably, most studies using murine models of aging or neurodegeneration have focused on males or mixed‐sex cohorts, leaving sex‐specific differences in neurogenesis and cognitive decline largely unexplored. Recent evidence indicates that diterpenoid treatment is associated with improved cognitive performance and enhanced neurogenesis in 6‐month‐old male SAMP8 mice. However, whether females exhibit similar responses remains unknown. In this study, we characterized sex differences in hippocampal neurogenesis in 6‐month‐old SAMP8 mice and examined potential sex‐dependent effects of diterpenoid therapy. Our findings reveal sex‐specific differences in physiological and pathological hippocampal neurogenesis and in responsiveness to ER272 treatment. Male SAMR1 mice displayed higher baseline neurogenesis than females, while male and female SAMP8 mice followed distinct neurogenic aging trajectories. ER272 was associated with improved cognitive performance and preservation of multiple neurogenic parameters in males but showed limited effects in females. These results suggest sex‐specific mechanisms of brain aging and indicate that mixed‐sex analyses may mask biologically relevant differences, highlighting the importance of considering sex in aging studies and therapeutic development.

## Graphical abstract

Male and female SAMP8 mice exhibit different physiological and pathological trajectories of hippocampal neurogenesis despite exhibiting similar cognitive performance. ER272 treatment was associated with improved cognition and enhanced neurogenesis exclusively in males highlighting the importance of sex‐specific analysis.

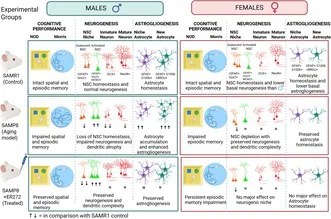

## 1 Introduction

Aging is associated with a progressive decline in hippocampal neurogenesis (Babcock et al. 2021; Gomez‐Oliva et al. 2023; Ibrayeva et al. 2021), which has been correlated with cognitive deterioration and neurodegenerative disorders such as Alzheimer's disease (Liu et al. 2023; Disouky et al. 2026; Terreros‐Roncal et al. 2021). Several murine models have been used so far to study the effects of aging on cognitive performance and neurodegeneration. The SAMP8 mouse model, characterized by accelerated aging and cognitive deficits (Ong et al. 2025), has been widely used to study age‐related hippocampal dysfunction (Diaz‐Moreno et al. 2018). Besides exhibiting multiple signs of aging, this model displays an early‐onset and progressive decline in cognitive function, which becomes evident around 4 months of age (Yagi et al. 1988). From 6 months onward, SAMP8 mice begin to show neuropathological symptoms commonly associated with Alzheimer's disease (Butterfield and Poon 2005). Notably, an initial increase in neurogenic activity in the dentate gyrus of the hippocampus (DG) occurs during early stages, which ultimately leads to a depletion of the neural stem cell (NSC) pool. This exhaustion appears to be driven by extrinsic signals within the neurogenic niche (Diaz‐Moreno et al. 2013; Gang et al. 2011; Soriano‐Canton et al. 2015).

Recent findings suggest that cognitive resilience in aging is closely linked to the functional responsiveness of neurons generated in the DG during adulthood (Montaron et al. 2020). This study indicates that memory performance in older individuals depends not only on the quantity of adult‐born neurons, but also on how effectively these neurons are activated and integrated into cognitive processes (Montaron et al. 2020). However, most studies describing the relevant role of neurogenesis in neuropathological aging have been performed in male subjects (Diaz‐Moreno et al. 2018; Diaz‐Moreno et al. 2013; Gomez‐Oliva et al. 2023) or using subjects from both sexes without analyzing the influence of sex (Liu et al. 2023). Thus, sex differences in neurogenesis and its response to stimuli remain poorly understood, despite increasing evidence that aging and neurodegeneration differ between males and females (Lee et al. 2025; Yagi et al. 2020). Notably, recent studies have identified sex‐specific differences in adult hippocampal neurogenesis. Males show a faster maturation and greater attrition of newly generated neurons in the DG compared to females (Yagi et al. 2020), suggesting that neurogenesis in males may be more sensitive to aging (Chow et al. 2013). These findings emphasize the importance of incorporating sex as a key biological variable in experimental research on neuroplasticity and cognitive aging.

Understanding the impact of sex on DG plasticity is essential for developing effective therapeutic strategies; however, sex‐specific responses to therapeutic interventions are understudied aspects of neurobiology (Hyer et al. 2018). Many preclinical studies fail to include both sexes, yet pharmacological and lifestyle interventions often yield different outcomes in males and females (Knufinke et al. 2023; Waters et al. 2021). This is particularly relevant in aging research, where therapies aimed at enhancing neurogenesis or slowing cognitive decline may be differentially effective depending on biological sex. Identifying these differences could lead toward sex‐specific treatments adapted to the individual aging trajectories of male and female individuals.

A recent report shows that two‐month treatment with diterpene ER272 ameliorates cognitive decline in 6‐month‐old male SAMP8 mice by enhancing hippocampal neurogenesis (Gomez‐Oliva et al. 2023). ER272 is a non‐tumor‐promoting 12‐deoxyphorbol diterpene isolated from plants of the Euphorbia genus that activates classical protein kinase C (PKC) isoforms through interaction with the C1B regulatory domain (Ezzanad et al. 2021; Geribaldi‐Doldan et al. 2015). Previous studies have shown that ER272 promotes TGF‐α release and exerts neurogenic and neuroprotective effects in experimental models (Dominguez‐Garcia et al. 2021; Gomez‐Oliva et al. 2023). Despite the observed effects, it is uncertain whether such treatment produces similar effects in females. In this study, we aimed to characterize sex differences in hippocampal neurogenesis and cognitive performance in 6‐month‐old SAMP8 mice and to determine whether diterpenoid treatment responses differ between sexes. Our findings provide novel insights into how sex influences hippocampal neurogenesis and cognition in aging, and highlight the relevance of considering biological sex in the design of therapeutic strategies for age‐related cognitive decline.

## 2 Methodology

### 2.1 Compound and Treatment

The isolation and purification of 12‐deoxyphorbol 13‐isobutyrate, also known as DPB (Ezzanad et al. 2021) or ER272 (Dominguez‐Garcia et al. 2021) (CAS: 25090‐74‐8) were carried out in our laboratory according to the procedures described in the supplementary methods of Geribaldi‐Doldan et al. (Geribaldi‐Doldan et al. 2015).

### 2.2 Animal Subjects

Male and female SAMP8/TaHsd and SAMR1/TaHsd mice were obtained from Envigo (currently Inotiv, The Netherlands). Mice aged four months were housed in a temperature‐controlled environment (21°C–23°C) with a 12‐h light/dark cycle and had ad libitum access to standard chow (AO4 maintenance diet, SAFE, Épinay‐sur‐Orge, France) and water. All procedures involving animals adhered to the European Union Directive (2010/63/EU) and the Spanish legal frameworks (65/2012 and RD53/2013) regulating the use of animals in research. Experiments were reported in accordance with the ARRIVE guidelines (Kilkenny et al. 2010).

The estrous cycle of female mice was not monitored during the experimental period. Given the two‐month chronic treatment, transient hormonal variations associated with the cycle were considered unlikely to exert a consistent or directional influence on the measured outcomes. Moreover, regular monitoring of the estrous cycle would have introduced additional handling‐related stress, potentially confounding behavioral and molecular analyses. This approach is consistent with previous studies using long‐term interventions in female rodents in which the estrous stage was not a primary experimental variable (Prendergast et al. 2014).

Animals were age‐matched and maintained under identical housing conditions. Due to the large number of experimental groups required for the study, littermate controls were not systematically used. The number of animals used varied among the different experiments and is given in each figure legend.

### 2.3 Protocol, Experimental Strategy, Treatments, and Groups

Four‐month‐old male and female SAMP8 mice were treated with either vehicle or ER272 every day for eight consecutive weeks. Mice were sacrificed at the end of the treatment (see Figure 1A for a schematic representation of the experimental workflow). Behavioral testing took place in the last 2 weeks before sacrifice and lasted for 10 days, with a 2‐day interval between open field‐NOD and MWM test, accounting for a total of 12 days. Age‐matched SAMR1 mice of both sexes were included as controls as shown in previous studies (Takeda 2009). In order to label newly generated cells, throughout the treatment period, all animals received intraperitoneal injections of 5‐bromo‐2′‐deoxyuridine (BrdU) every other day (75 mg/kg) (see Figure 1A for experimental design). The SAMR1 strain was used as a reference group to distinguish between baseline values and those related to the pathological features of the SAMP8 aging model. ER272 was administered exclusively to SAMP8 mice in order to evaluate its potential to prevent the cognitive and neurogenic impairments characteristic of this model. Similar experimental approaches have been previously reported (Iwata et al. 2020; Shi et al. 2020).

**FIGURE 1:**
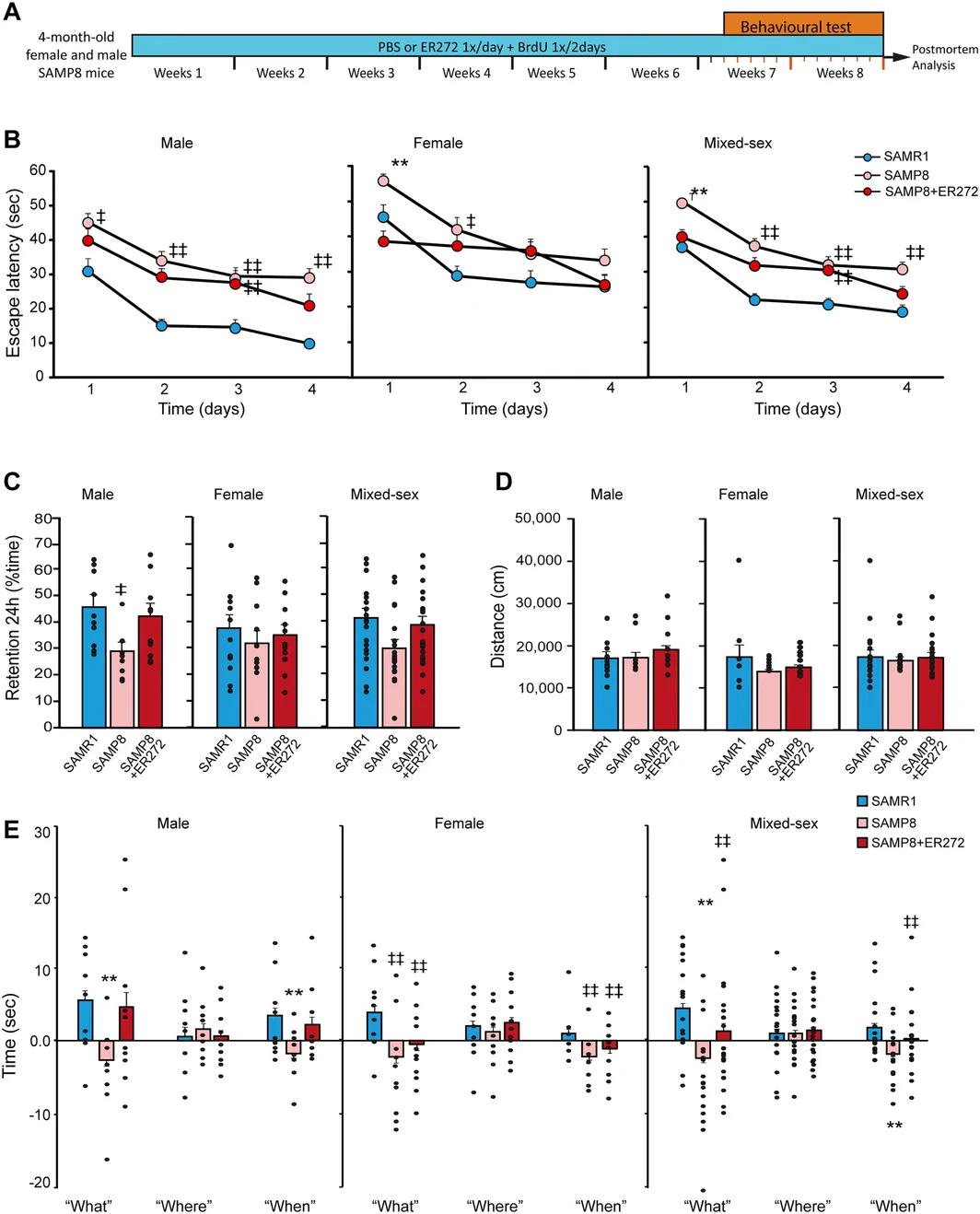
Treatment of SAMP8 mice with ER272 improves cognitive performance in male but not in female. (A) Experimental design. Four‐month‐old male and female SAMP8 mice were treated with vehicle or ER272 (SAMP8 + ER272) for 8 weeks until the age of 6 months. They were compared with SAMR1 mice of the same age treated with vehicle. Mice received BrdU during the 8‐week period every 2 days. Behavior tests took place during the last 12 days. (B) Scape latency (sec) in the MWM test. (Left) An impairment was observed in SAMP8 male mice when they were evaluated in the MWM and a slight improvement was observed for SAMP8‐ER mice. No significant group X day effect was detected [*F*(2,462) = 0.358, *p* = 0.905], comparisons at individual training days revealed improved performance in ER272‐treated SAMP8 mice (Day 1 [*F*(2,116) = 4.64, ‡*p* = 0.031 vs. SAMR1], Day 2 [*F*(2,116) = 10.26, ‡‡*p* < 0.01 vs. SAMR1], Day 3 [*F*(2,113) = 3.67, ‡‡*p* < 0.001 vs. SAMR1], Day 4 [*F*(2,116) = 11.14, ‡‡*p* < 0.001 vs. SAMR1]). Data are representative of 10 mice (SAMR1 *n* = 10, SAMP8 *n* = 10, SAMP8‐ER *n* = 10). (Center) An impairment was observed in SAMP8 female mice when they were evaluated in the MWM and a slight improvement was observed for SAMP8‐ER mice. No significant group X day effect was detected [*F*(2,495) = 2.02, *p* = 0.061], however individual daily assessment revealed a better performance as training sessions progressed (Day 1 [*F*(2,125) = 8.75, **p < 0.001 vs. rest of the groups], Day 2 [*F*(2,126) = 3.90, ‡*p* = 0.023 vs. SAMR1], Day 3 [*F*(2,115) = 1.97, *p* = 0.143], Day 4 [*F*(2,129) = 1.73, *p* = 0.180]. Data are representative of 10–12 mice (SAMR1 *n* = 10, SAMP8 *n* = 11, SAMP8‐ER *n* = 12). (Right) An impairment was observed in SAMP8 mice when male and female cohorts were evaluated together in the MWM and a slight improvement was observed for SAMP8‐ER mice. No significant group X day effect was detected [*F*(2,969) = 1.898, *p* = 0.078], however individual daily assessment revealed a better performance as training sessions progressed (Day 1 [*F*(2,244) = 9.21,**p < 0.001 vs. rest of the groups], Day 2 [F(2,245) = 12.84, ‡‡*p* < 0.001 vs. SAMR1], Day 3 [F(2,231) = 7.98, ‡‡*p* < 0.001 vs. SAMR1], day 4 [*F*(3,249) = 9.02, ‡‡*p* < 0.001 vs. SAMR1]. Data are representative of 20–22 mice (SAMR1 *n* = 20, SAMP8 *n* = 21, SAMP8‐ER *n* = 22). (C) Retention test in the MWM. In the 24 h retention phase SAMP8 male mice (left) spent shorter times in quadrant 2 where the platform used to be located. [*F*(2,25) = 4.16, ‡*p* = 0.028 vs. SAMR1] (SAMR1 *n* = 10, SAMP8 *n* = 10, SAMP8‐ER *n* = 10) No differences were detected among females (center) [*F*(2,29) = 0.288, *p* = 0.752] (SAMR1 *n* = 10, SAMP8 *n* = 11, SAMP8‐ER *n* = 11). No differences were observed in the retention phase when both cohorts were compared together (right) 24 h [*F*(2,58) = 2.51, *p* = 0.09] after completing the acquisition phase (SAMR1 *n* = 20, SAMP8 *n* = 19, SAMP8‐ER *n* = 21). (D) Distances traveled in the open field. No differences were observed among groups when distances traveled in the open field were analyzed in males [*F*(3,27) = 0.717, *p* = 0.497], females [*F*(2,24) = 0.89, *p* = 0.420] or both cohorts together [*F*(2,54) = 0.27, *p* = 0.763]. Data are representative of 7–12 mice (SAMR1 *n* = 10, SAMP8 *n* = 10, SAMP8‐ER *n* = 10; ♀: SAMR1 *n* = 8, SAMP8 *n* = 12, SAMP8‐ER *n* = 7). Sex comparisons: Motor activity (total distance traveled) in males and females in the groups under study (SAMR1 [*F*(1,16) = 0.845, *p* = 0.372], SAMP8 [*F*(1,15) = 3.55, *p* = 0.560], SAMP8‐ER [*F*(1,20) = 1.40, *p* = 0.250]). Differences detected by one‐way ANOVA followed by Tuckey b test. (E) NOD test. (Left) Episodic memory was severely affected in male SAMP8 mice when “what” and “when” paradigms were assessed, while ER treatment counterbalanced this situation. “What” [*F*(2,84) = 9.55, **p < 0.001 vs. rest of the groups] (SAMR1 *n* = 10, SAMP8 *n* = 9, SAMP8‐ER *n* = 10), “where” [*F*(2,84) = 0.309, *p* = 0.735] (SAMR1 *n* = 9, SAMP8 *n* = 10, SAMP8‐ER *n* = 10), “when” [*F*(2,89) = 8.69, **p < 0.001 vs. rest of the groups] (SAMR1 *n* = 10, SAMP8 *n* = 10, SAMP8‐ER *n* = 10). (Center) Episodic memory was severely affected in female SAMP8 mice when “what” and “when” paradigms were assessed, while ER treatment improved this impairment, differences did not reach statistical significance. “What” [*F*(2,93) = 15.60, ‡‡*p* < 0.001 vs. SAMR1] (SAMR1 *n* = 10, SAMP8 *n* = 11, SAMP8‐ER *n* = 11), “where” [*F*(2,96) = 1.11, *p* = 0.332] (SAMR1 *n* = 10, SAMP8 *n* = 11, SAMP8‐ER *n* = 12), “when” [*F*(2,90) = 8.81, ‡‡*p* < 0.001 vs. SAMR1] (SAMR1 *n* = 10, SAMP8 *n* = 11, SAMP8‐ER *n* = 10). (right) Episodic memory was severely affected in SAMP8 mice when “what” and “when” paradigms were assessed in both male and female cohorts together, while ER treatment counterbalanced this situation. “What” [*F*(2,180) = 20.63, **p < 0.001 vs. rest of the groups, ‡‡*p* < 0.001 vs. SAMR1] (SAMR1 *n* = 20, SAMP8 *n* = 20, SAMP8‐ER *n* = 21), “where” [*F*(2,183) = 0.132, *p* = 0.876] (SAMR1 *n* = 20, SAMP8 *n* = 21, SAMP8‐ER *n* = 22), “when” [*F*(2,180) = 15.33, **p < 0.001 vs. rest of the groups, ‡‡*p* < 0.001 vs. SAMR1] (SAMR1 *n* = 20, SAMP8 *n* = 21, SAMP8‐ER *n* = 22). Sex comparisons: In the NOD test SAMP8‐ER females showed worse responses in the “what” and “when” paradigms than males (what: SAMR1 [*F*(1,58) = 0.541, *p* = 0.465], *p* = 0.898], SAMP8 [*F*(1,58) = 0.030, *p* = 0.864], SAMP8‐ER [*F*(1,61) = 7.161, *p* = 0.01]; where: SAMR1 [*F*(1,55) = 1.265, *p* = 0.266], SAMP8 [*F*(1,61) = 0.100, *p* = 0.753], SAMP8‐ER [*F*(1,64) = 3.776, *p* = 0.056]; when: SAMR1 [*F*(1,58) = 2.456, *p* = 0.068], *p* = 0.732], SAMP8 [*F*(1,61) = 0.836, *p* = 0.364], SAMP8‐ER [*F*(1,58) = 8.305, *p* = 0.006]).

### 2.4 Intranasal Delivery of ER272

The compound ER272 was administered intranasally with the animals in vertical position and their necks gently extended, following procedures described by Marks et al. (Marks et al. 2009). A total of 18 μL ER272 solution (1 μM in 0.9% saline) or saline was dispensed into the nostrils, alternating 3 μL per nostril using a micropipette. Mice remained in this position for approximately 10 s post‐administration to promote full nasal absorption. Care was taken to minimize potential spillage by administering the solution in small aliquots while maintaining the animals in a vertical position before and after administration. No obvious leakage was observed during the procedure. Treatment assignment (vehicle or ER272) was randomized and coded, and all outcome measures were evaluated by investigators blinded to group allocation.

### 2.5 New Object Discrimination Test (NOD)

Behavioral testing began 12 days before the end of the study. The habituation phase began with the exposure to two different objects (a red cylinder and a yellow trapezoid). One day after the habituation phase, we assessed integrated episodic memory for the paradigms “what,” “when,” and “where” (Ramos‐Rodriguez et al. 2013). On day one, mice explored the habituation objects. On the following day, they encountered four copies of a new object (blue balls) arranged in a triangular pattern for 5 min. After 30 min, a second exposure included four novel items (red cones) in a square configuration. The final test phase, conducted 30 min later, involved two “recent” objects from the second exposure (in unchanged positions) and two “familiar” objects from the first exposure, once in the original location and one relocated. Memory components were quantified as: “what” (exploration time difference between familiar and recent objects), “where” (difference in time spent with displaced vs. non‐displaced objects), and “when” (preference between familiar and recent items in original positions), following previously established experimental designs (Dere et al. 2005; Infante‐Garcia et al. 2018; Segado‐Arenas et al. 2018).

### 2.6 Morris Water Maze (MWM)

Forty‐eight hours after completing the NOD task, spatial learning and memory performance were evaluated using the MWM test in all experimental groups, based on the procedure described by Ramos‐Rodriguez et al. (Ramos‐Rodriguez et al. 2013). In brief, the Morris water maze consisted of a circular tank measuring 95 cm in diameter, virtually divided into four quadrants. Visual cues in the form of geometric figures were placed around the pool to aid spatial orientation. The escape platform was positioned in quadrant 2 and submerged 2 cm below the water surface, with water maintained at 21°C ± 1°C. The acquisition phase spanned four consecutive days, during which each mouse underwent four trials per day, spaced 10 min apart. The starting point changed randomly along the acquisition phase. Each trial had a maximum duration of 60 s, and mice were allowed to remain on the platform for 10 s upon reaching it. If the animal failed to locate the platform within the allotted time, it was gently placed on it for the same duration. 24 h after the last training session, a probe test was conducted: the platform was removed, and mice were allowed to swim freely for 60 s. Escape latency during the acquisition trials and the time spent in the target quadrant and in other quadrants during the probe test were measured and analyzed using SMART software (Panlab, Spain).

### 2.7 Open Field

Spontaneous motor activity was analyzed before NOD test by measuring the distance traveled by mice for 30 min in a rectangular box (22 × 44 × 40 cm), as described (Infante‐Garcia et al. 2017). The total distance traveled and the velocity were recorded.

### 2.8 Tissue Processing and Immunohistochemistry

At the end of treatments, mice were administered Dolethal (Ventoquinol, Lure, France) containing a lethal 50 mg dose of pentobarbital and perfused with 4% paraformaldehyde (PFA) via ascending aorta. Brains were sectioned at 30 μm thickness using a cryostat. Immunostaining followed protocols described in earlier publications (Garcia‐Bernal et al. 2018; Geribaldi‐Doldan et al. 2015; Murillo‐Carretero et al. 2017). Detailed antibody information is provided in Tables S1 and S2.

### 2.9 Quantification of Hippocampal Neurogenesis

BrdU, doublecortin (DCX), NeuN, glial fibrillary acidic protein (GFAP), SRY‐box transcription factor 2 (SOX2), and S100 calcium‐binding protein B (S100β)‐positive cells in the DG were quantified as previously described (Rabaneda et al. 2008; Rabaneda et al. 2016). After perfusion, brains were anonymized with coded labels. Cell counts were performed blindly on every fifth coronal section encompassing the hippocampus (14–16 sections per animal), using a Zeiss LSM 900 Airyscan 2 confocal microscope. Z‐stacks were acquired every 0.8 μm with a 20X objective. Cell density values were normalized to the DG volume and averaged per animal, as described by Dominguez‐Garcia et al. (Dominguez‐Garcia et al. 2021).

### 2.10 Morphometric Analysis of DCX‐Positive Neurons

Three‐dimensional reconstructions of DCX‐labeled neurons were obtained from confocal image stacks using Neurolucida 360 software (MBF Bioscience, VT, USA). Morphological parameters, including total dendritic length, surface area, segment number, terminal branches, and Sholl intersections, were quantified using Neurolucida Explorer (Carrascal et al. 2009; Nunez‐Abades et al. 1994).

### 2.11 Statistical Approach

All statistical analyses followed pharmacological research guidelines for rigorous experimental design (Fountain et al. 2014). Analyses were conducted in IBM SPSS Statistics v22. Data normality was tested using the Shapiro–Wilk test, followed by Brown‐Forsythe's test for homogeneity of variances. One‐way ANOVA with Tukey's post hoc correction was used for multi‐group comparisons. For the MWM acquisition phase, two‐way ANOVA (group × day) was applied. Significance was set at *p* < 0.05. Sample sizes ranged from *n* = 6–10 (in postmortem studies), and *n* = 10–12 (behavioral studies), consistent with prior work (Carrasco et al. 2014; Dominguez‐Garcia et al. 2020; Geribaldi‐Doldan et al. 2018; Rabaneda et al. 2016). The exact number of replicates (*n*) for each experiment is provided in the corresponding figure legends and summarized in Table S5, which contains the complete statistical output, including sample sizes and exact *p* values for all comparisons, regardless of statistical significance. Minor variations in sample size between analyses reflect endpoint‐specific technical requirements and predefined quality‐control criteria applied during behavioral scoring, tissue processing, immunohistochemistry, and neuronal reconstruction.

## 3 Results

### 3.1 Sex‐Specific Efficacy of Diterpenoid Treatment: Cognitive Recovery in Male but Not Female SAMP8 Mice

To test whether adult hippocampal neurogenesis and cognitive performance differ between male and female SAMP8 mice and to determine their responsiveness to ER272 treatment, we used the experimental approach represented in Figure 1A. Four‐month‐old male and female SAMP8 mice were treated with either vehicle or ER272 during eight consecutive weeks. Male and female SAMR1 mice were used as the control group according to previous reports (Takeda 2009). Twelve days before treatment completion, animals underwent behavioral testing (MWM, NOD, and open field). Finally, mice were euthanized at the end of the 8‐week treatment period once behavioral tests were completed.

Spatial memory was analyzed in six‐month‐old SAMP8 male and female mice after treatment with diterpene ER272, or vehicle. An impairment was observed in SAMP8 male and female mice when they were evaluated in the training phase of the MWM and a slight improvement was observed for SAMP8 male and female mice treated with ER272 when analyzing the entries in quadrant 2, where the platform was located (Figure 1B). No differences were observed when we compared the time males or females spent in quadrants 1, 3 and 4 (see Table S4). In addition, no significant group × day interaction was detected, indicating a similar rate of learning across groups. However, comparisons at individual training days revealed improved performance in ER272‐treated SAMP8 mice, with escape latencies that became comparable to those of SAMR1 mice as training progressed. In the retention phase, SAMP8 male mice showed a statistically significant reduction in the time spent in the platform quadrant (Figure 1C). This reduction was not observed in female mice (Figure 1C). Moreover, treatment with ER272 improved retention in male mice, whereas no differences were detected in female SAMP8 mice treated with ER272 compared to female SAMP8 treated with vehicle (Figure 1C). When we compared males and females from different groups in the acquisition phase of the MWM we did not detect a significant day × sex effect in any of the groups under study, although significant differences were observed for day and sex criteria (Table S3). On the other hand, sex comparisons in the retention phase 24 h after training did not reveal significant differences in any of the groups under study. Similarly, no differences were detected when the number of entries into quadrant 2 were compared between sexes in all groups under study. As control, no differences were found in any of the groups when the distance traveled in the open field was analyzed (Figure 1D) and neither when male and female were compared.

Episodic memory was tested in the NOD test. Results showed that episodic memory was severely affected in male and female SAMP8 mice when “what” and “when” paradigms were analyzed (*p* < 0.001). Treatment with ER272 rescued this situation only in male mice whereas no improvement was observed in female in response to the treatment (Figure 1E). When sexes were compared in the NOD test we observed that SAMP8‐ER females displayed worse responses in the “what” and “when” paradigms than males.

### 3.2 Sex‐Dependent Differences in Neurogenesis in the DG and Their Response to Treatment in Six‐Month‐Old SAMP8 Mice

To investigate neurogenesis in the DG (Figure 2) of SAMP8 mouse and assess the influence of sex and diterpene treatment, mice received BrdU injections every other day throughout the treatment period. As shown in Figure 2A,B, both male and female SAMP8 mice exhibited a reduction in the number of BrdU‐incorporating cells after 2 months of treatment, although this decrease did not reach statistical significance. Notably, treatment with ER272 led to a significant increase in BrdU^+^ cells in both sexes (Figure 2A,B). This effect was particularly marked in male SAMP8 mice, where the number of BrdU^+^ cells exceeded that observed in SAMR1 controls (Figure 2A,B). Interestingly, the number of BrdU^+^ cells in ER272‐treated males doubled that found in treated females (Figure 2A,B).

**FIGURE 2:**
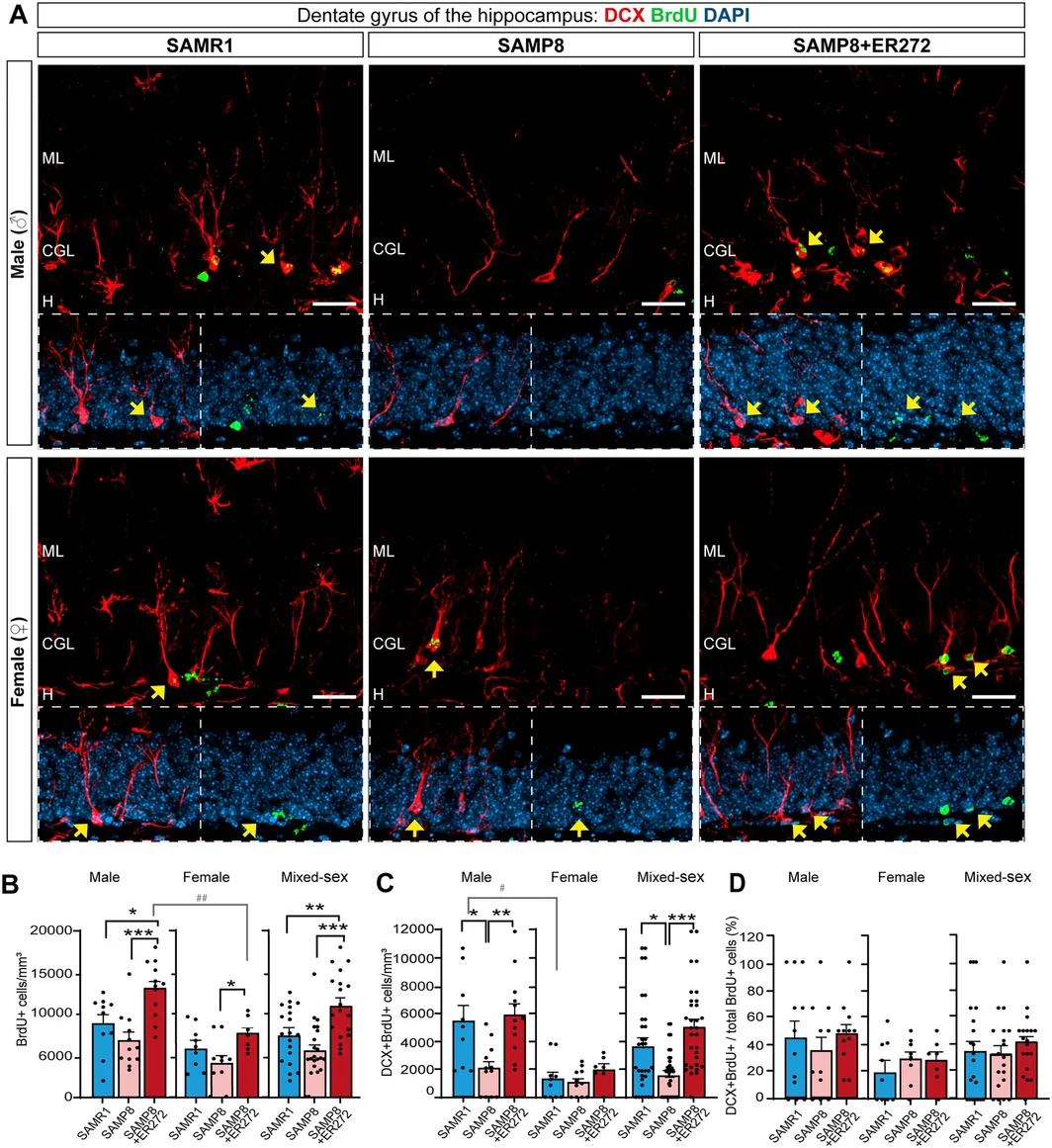
Two‐month intranasal administration of ER272 to SAMP8 mice differentially affects DCX^+^ neuroblasts in male and female. (A) Representative confocal microcopy images of the DG of the hippocampus of six‐month‐old SAMR1 (left) and SAMP8 male mice (center) treated with vehicle or SAMP8 mice treated with ER272 (SAMP8 + ER272) right in female (upper panel) and male (lower panel) mice. Tissue was processed for the detection of the proliferation marker BrdU and DCX. DAPI staining is shown in blue. Only merged channels are shown. Yellow arrows indicate DCX^+^BrdU^+^DAPI^+^ cells. (B) Graph shows the total number of BrdU^+^ nuclei in the DG of the hippocampus per mm^3^ in male (left) [*F*_(2,31)_ = 10.88, *p = 0.018 SAMR1 vs. SAMP8 + ER272] [*F*_(2,31)_ = 10.88, ***p = 0.0002 SAMP8 vs. P8 + ER272], female (center) [*F*_(2,23)_ = 3.85, *p = 0.03 SAMP8 vs. SAMP8 + ER272] and both cohorts together (right) [*F*_(2,57)_ = 12.47, **p = 0.01 SAMR1 vs. SAMP8 + ER272] [*F*_(2,57)_ = 12.47, ***p < 0.0001 SAMP8 vs. SAMP8 + ER272]. Sex differences in BrdU^+^ cells are also shown for SAMP8 + ER272 group [*F*_(5,54)_ = 11.2, # *p* = 0.005]. Sample size: SAMR1 (♂ *n* = 10, ♀ *n* = 9), SAMP8 (♂ *n* = 12, ♀ *n* = 10), SAMP8 + ER272 (♂ *n* = 12, ♀ *n* = 7). (C) Graph shows the total number of DCX^+^BrdU^+^ cells in the DG of the hippocampus per mm^3^ in male (left panel) [*F*_(2,30)_ = 7.387, *p = 0.02 SAMR1 vs. P8] [*F*_(2,30)_ = 7.387, **p < 0.003 SAMP8 vs. SAMP8 + ER272], female (center panel) [*F*(2,25) = 1.340, *p* = 0.280; no significant differences among groups] and both sexes together (left panel) [both cohorts together (right panel) [*F*(2,94) = 12.59, *p = 0.04 SAMR1 vs. SAMP8] [*F*(2,94) = 12.59, ***p < 0.0001 SAMP8 vs. SAMP8 + ER272]. Sex differences in DCX^+^BrdU^+^ cells are also shown for the SAMR1 group [*F*(5,55) = 10.75, # *p* = 0.016]. Sample size: SAMR1 (♂ *n* = 9, ♀ *n* = 10), SAMP8 (♂ *n* = 12, ♀ *n* = 11), SAMP8 + ER272 (♂ *n* = 12, ♀ *n* = 7). (D) No statistically significant differences were detected in the percentage of DCX^+^BrdU^+^ cells relative to total BrdU^+^ cells among groups or between sexes. Data are the means ± S.E.M. Sample size: SAMR1 (♂ *n* = 12, ♀ *n* = 9), SAMP8 (♂ *n* = 11, ♀ *n* = 9), SAMP8 + ER272 (♂ *n* = 12, ♀ *n* = 7). Differences detected by one‐way ANOVA followed by Tukey b test. Scale bar represents 25 μm.

The analysis of immature neurons generated over the course of the treatment (BrdU^+^DCX^+^), showed an increase in male SAMR1 by 5‐fold compared to SAMR1 females. Interestingly, male SAMP8 mice showed a significant reduction in DCX^+^BrdU^+^ cells, which was fully prevented by ER272 treatment (Figure 2A,C). In contrast, female SAMP8 mice did not differ from female SAMR1 controls in the number of newly generated DCX^+^BrdU^+^ cells. This result indicated that control male generated a larger number of new neurons from months 4 to 6 compared to female. On the contrary, the number of DCX^+^BrdU^+^ cells in SAMP8 male mice is comparable to that of control and SAMP8 female. Remarkably, the treatment prevents the reduction found in male whereas in treated female a slight increase was observed that was not statistically significant. The percentage of BrdU^+^ cells that had differentiated into DCX^+^ cells was also calculated (BrdU^+^DCX^+^/total BrdU^+^ cells (%)). As shown in Figure 2A,D, around 40% of all the BrdU^+^ cells differentiated into DCX^+^ cells in SAMR1 male mice. This percentage was around 20% in female, although this difference was not statistically significant. No statistically significant alterations were observed in SAMP8 mice compared to SAMR1 (male and female), nor an effect of the treatment (Figure 2A,D), suggesting that differentiation of BrdU^+^ cells in male SAMR1 mice might be facilitated in male compared to female, but no particular alteration was found in SAMP8 with and without treatment.

We next analyzed the number of newly generated mature neurons (NeuN^+^BrdU^+^ cells). Male of all groups displayed a higher number of NeuN^+^BrdU^+^ compared to female; however, these differences did not reach statistical significance in the case of SAMR1 mice. No statistically significant differences were found between SAMP8 and SAMR1 mice in either sex (Figure 3A,B). However, ER272 treatment significantly increased the number of newly generated mature neurons in male SAMP8 mice, a tendency that was also observed in females, but it was not statistically significant. Interestingly, the proportion of BrdU^+^ cells acquiring a mature neuronal phenotype remained unchanged across groups (Figure 3A,C), indicating that neither sex, disease or treatment is modulating the final neuronal fate of BrdU^+^ cells.

**FIGURE 3:**
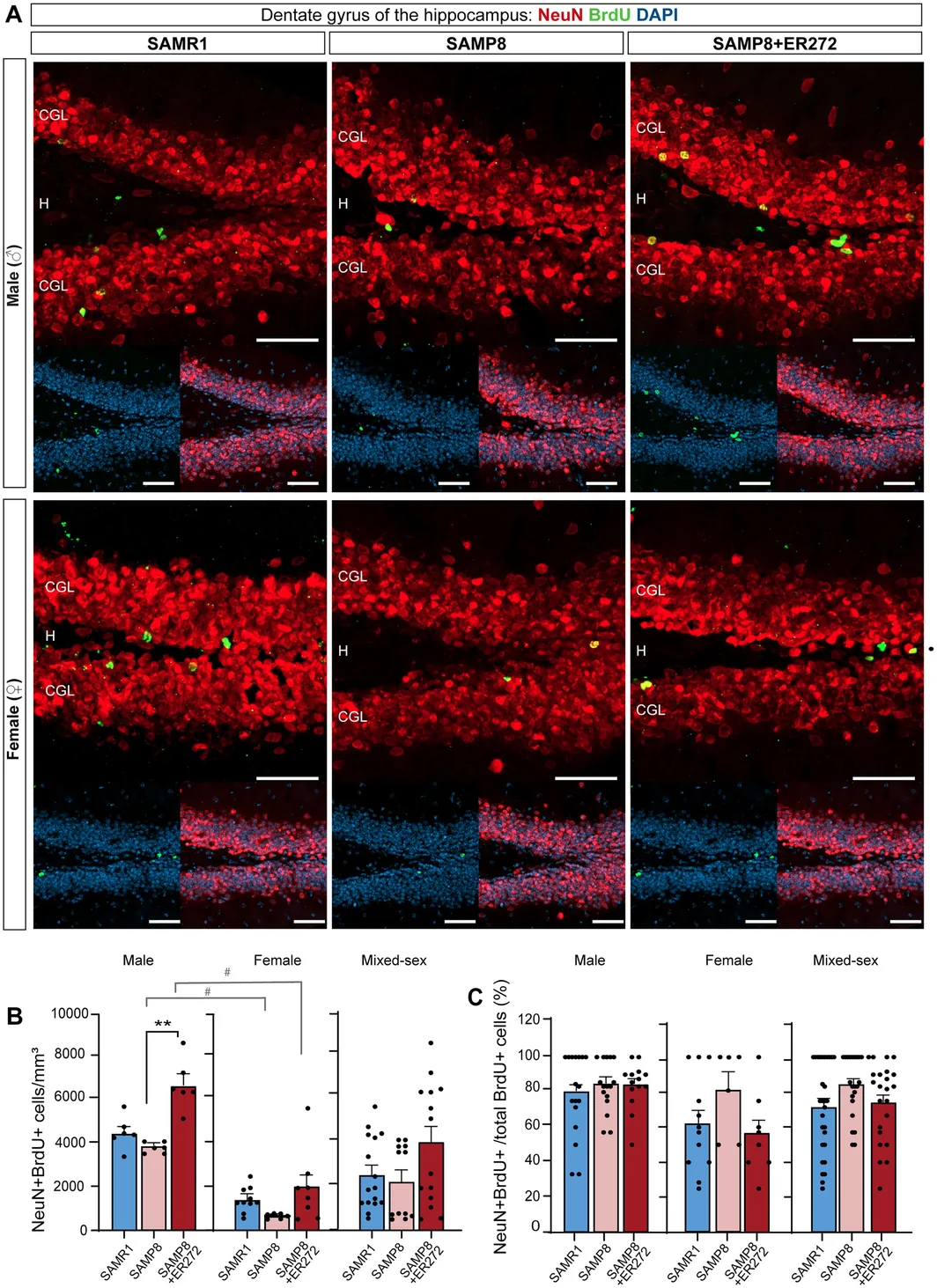
Sex dependent effect of intranasal administration of ER272 to SAMP8 mice on NeuN^+^ cells. (A) Representative confocal microcopy images of the DG of the hippocampus of six‐month‐old SAMR1 and SAMP8 male mice treated with vehicle or SAMP8 mice treated with ER272 during 8 weeks. Female (upper panel) and male (lower panel). Slices were processed for the immunohistochemical detection of the proliferation marker BrdU and NeuN. DAPI staining is shown in blue. Only merged channels are shown. Yellow arrows indicate NeuN^+^BrdU^+^DAPI^+^ cells. (B) Graph shows the total number of NeuN^+^BrdU^+^ cells in the DG of the hippocampus per mm^3^ in male (left panel) [*F*(2,15) = 20.25, **p = 0.0013 SAMP8 vs. SAMP8 + ER272], female (center panel) [*F*(2,21) = 2.826, *p* = 0.0819; no significant differences among groups] and both cohorts together (right panel) [*F*(2,39) = 2.598, *p* = 0.0872; no significant differences among groups]. Sex differences in NeuN^+^BrdU^+^ cells are also shown for the SAMP8 group [*F*(5,36) = 37.90, #*p* = 0.03] and the SAMP8 + ER272 group [*F*(5,36) = 37.90, #*p* = 0.011]. Sample size: SAMR1 (♂ *n* = 6, ♀ *n* = 10), SAMP8 (♂ *n* = 6, ♀ *n* = 6), SAMP8 + ER272 (♂ *n* = 6, ♀ *n* = 8). (C) No statistically significant differences were detected in the percentage of NeuN^+^BrdU^+^ cells relative to total BrdU^+^ cells among groups or between sexes. Sample size: SAMR1 (♂ *n* = 17, ♀ *n* = 12), SAMP8 (♂ *n* = 18, ♀ *n* = 6), SAMP8 + ER272 (♂ *n* = 14 ♀ *n* = 8). Data are expressed as mean ± S.E.M. Differences were analyzed by one‐way ANOVA followed by Tukey's multiple comparisons test. Scale bar represents 50 μm.

### 3.3 Sex‐Specific Alterations of the Complexity of Newly Generated Neurons Within the Neurogenic Niche and Treatment Response in SAMP8 Mice

The morphological properties of immature neurons were then analyzed across all experimental groups. No differences were observed in total dendritic length between male and female SAMR1 control mice (~200 μm) (Figure 4A,B). Male SAMP8 mice, however, showed a reduced dendritic length compared to SAMR1 controls, an effect that was prevented by ER272 treatment (Figure 4A,B). In contrast, SAMP8 females showed neither a reduction in dendritic length nor an effect of the treatment (Figure 4A,B). Dendritic surface area was also reduced in male SAMP8 mice relative to SAMR1 males, whereas no changes were detected in females. ER272 administration prevented this reduction in males (Figure 4A,C). Similarly, the number of dendritic segments and the number of terminal segments were reduced in male SAMP8 mice compared to SAMR1 but not in females (Figure 4A,D,E). Treatment with ER272 prevented this reduction in males whereas no effect of the treatment was observed in females (Figure 4A,D,E). In addition, segment order analysis showed a reduction in the number of quaternary and quinary segments in males that was prevented by ER272 in SAMP8 male mice. No statistically significant differences or treatment effect were detected in females (Figure 4F,G). Finally, Sholl analysis showed a lower number of intersections at 60–70 μm from the soma in male SAMP8 mice compared to SAMR1 controls (Figure 4A,G,H), an effect that was reverted by ER272 treatment. Interestingly, no differences were observed in females (Figure 4A,G,H). In general, no alterations in the morphology of DCX^+^ cells were found in females of all groups whereas male SAMP8 mice showed alterations that were reverted by the treatment.

**FIGURE 4:**
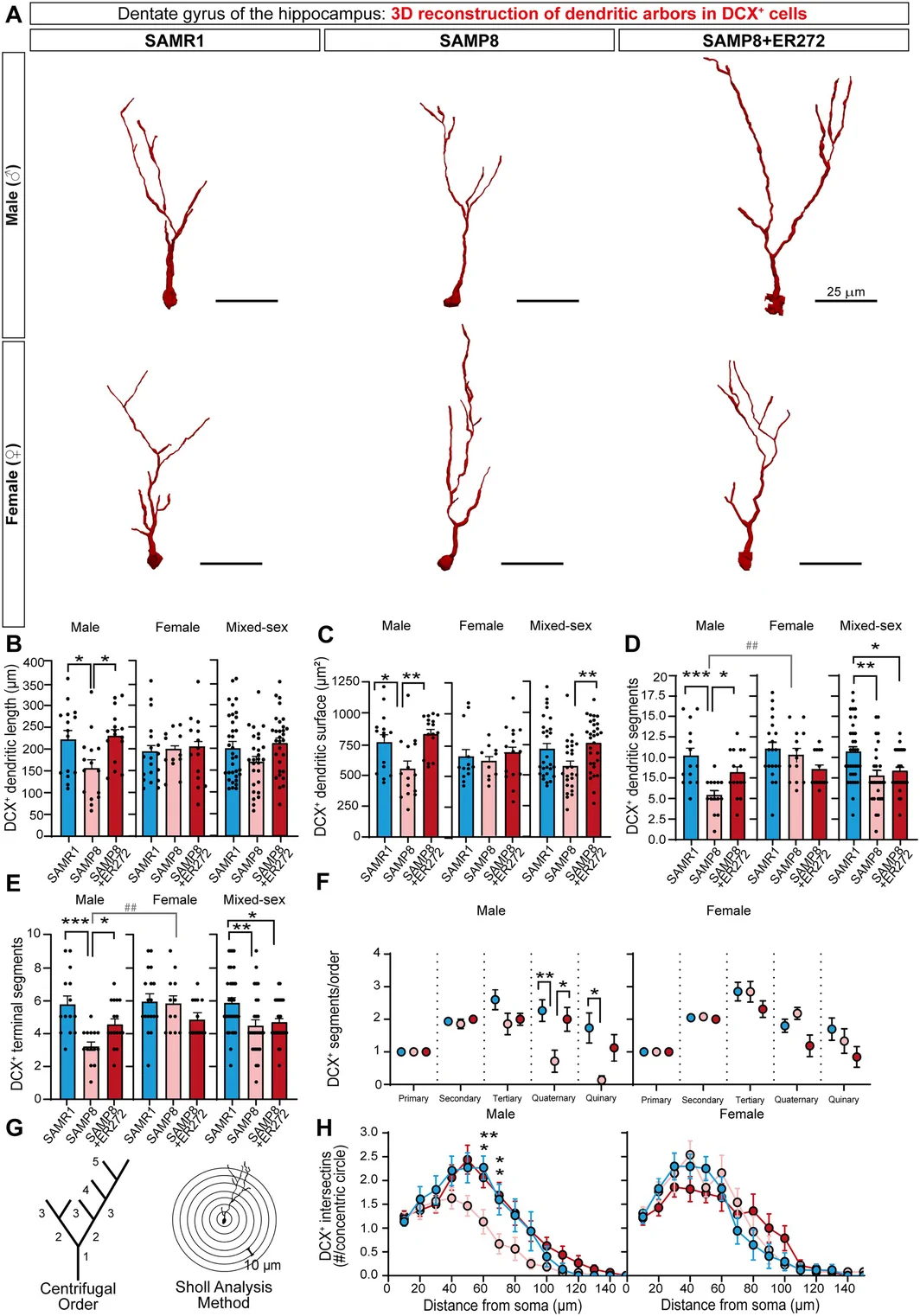
Sex dependent alteration of the morphology of DCX^+^ cells in SAMP8 and differential response to the treatment. (A) Examples of the reconstructed DCX^+^ cells used in the analysis of SAMR1 and SAMP8 treated with vehicle and SAMP8 treated with ER272. Female (upper panel) and male (lower panel). (B) Total dendritic length of DCX^+^ cells in males (left panel) [*F*(2,43) = 4.94, *p = 0.04 SAMR1 vs. SAMP8] [*F*(2,43) = 4.94, *p = 0.020 SAMP8 vs. SAMP8 + ER272], females (center panel) [*F*(2,42) = 0.036, *p* = 0.964; no significant differences among groups], and both cohorts together (right panel) [*F*(2,88) = 2.724, *p* = 0.071; no significant differences among groups]. No sex differences were detected in total dendritic length [*F*(5,85) = 1.787, *p* = 0.124]. Sample size: SAMR1 (♂ *n* = 15, ♀ *n* = 19), SAMP8 (♂ *n* = 15, ♀ *n* = 12), SAMP8 + ER272 (♂ *n* = 16, ♀ *n* = 14). (C) Total dendritic surface of DCX^+^ cells in males (left panel) [*F*(2,43) = 7.157, *p = 0.024 SAMR1 vs. SAMP8] [*F*(2,43) = 7.157, **p = 0.002 SAMP8 vs. SAMP8 + ER272], females (center panel) [*F*(2,35) = 0.240, *p* = 0.788; no significant differences among groups], and both cohorts together (right panel) [*F*(2,81) = 4.952, **p = 0.003 SAMP8 vs. SAMP8 + ER272]. No sex differences were detected in total dendritic surface. Sample size: SAMR1 (♂ *n* = 15, ♀ *n* = 13), SAMP8 (♂ *n* = 15, ♀ *n* = 10), SAMP8 + ER272 (♂ *n* = 16, ♀ *n* = 15). (D) Number of dendritic segments of DCX^+^ cells in males (left panel) [*F*(2,40) = 9.51, ***p < 0.001 SAMR1 vs. SAMP8] [*F*(2,40) = 9.51, *p = 0.040 SAMP8 vs. SAMP8 + ER272], females (center panel) [*F*(2,44) = 2.536, *p* = 0.091; no significant differences among groups], and both cohorts together (right panel) [*F*(2,87) = 6.846, **p = 0.003 SAMR1 vs. SAMP8] [*F*(2,87) = 6.846, *p = 0.018 SAMR1 vs. SAMP8 + ER272]. Sex differences in dendritic segments are also shown for the SAMP8 group [*F*(5,84) = 6.784, ##*p* = 0.0011]. Sample size: SAMR1 (♂ *n* = 14, ♀ *n* = 20), SAMP8 (♂ *n* = 14, ♀ *n* = 13), SAMP8 + ER272 (♂ *n* = 15, ♀ *n* = 14). (E) Number of terminal segments of DCX^+^ cells in males (left panel) [*F*(2,41) = 10.5, ***p < 0.001 SAMR1 vs. SAMP8] [*F*(2,41) = 10.5, *p = 0.050 SAMP8 vs. SAMP8 + ER272], females (center panel) [*F*(2,40) = 1.573, *p* = 0.220; no significant differences among groups], and both cohorts together (right panel) [*F*(2,84) = 6.379, **p = 0.004 SAMR1 vs. SAMP8] [*F*(2,84) = 6.379, *p = 0.03 SAMR1 vs. SAMP8 + ER272]. Sex differences in terminal segments are also shown for the SAMP8 group [*F*(5,81) = 6.508, ##*p* = 0.002]. Sample size: SAMR1 (♂ *n* = 14, ♀ *n* = 18), SAMP8 (♂ *n* = 14, ♀ *n* = 12), SAMP8 + ER272 (♂ *n* = 16, ♀ *n* = 13). (F, G) Number of segments per order of females, males (Quaternary order [*F*_(2,42)_ = 5.50, **p < 0.009 SAMR1 vs. SAMP8] [*F*_(2,42)_ = 5.50, *p = 0.032 SAMP8 vs. SAMP8 + ER272]; Quinary order [*F*_(2,43)_ = 4.76, *p < 0.011 SAMR1 vs. SAMP8]). (G, H) Sholl dependent analysis of females and males (60 μm from soma [*F*_(2,43)_ = 6.292, **p < 0.005 SAMR1 vs. SAMP8; *p < 0.022 SAMP8 vs. SAMP8 + ER272]; 70 μm from soma [*F*_(2,42)_ = 4.917, *p < 0.026 SAMR1 vs. SAMP8; *p < 0.025 SAMP8 vs. SAMP8 + ER272]). Scale bar represents 25 μm.

### 3.4 Differential Impact of Sex on Niche Astrocytes and Treatment Efficacy in Middle‐Aged SAMP8 Mice

Disproportionate astrogliogenesis in the DG niche is a characteristic of brain aging (Encinas et al. 2011). To assess whether sex had an impact on these alterations, we analyzed astrogliogenesis in senescent mice and evaluated the effect of the ER272 diterpene treatment on the generation of new astrocytes. As expected, a larger number of niche astrocytes (S100β^+^ cells) was found in male SAMP8 mice compared to SAMR1. This increase in niche astrocyte number was not observed in treated male mice (Figure 5A,B) although no changes were observed in females of all groups. The number of niche astrocytes in female mice of all groups was 2‐fold smaller than that of males (Figure 5A,B). The number of astrocytes generated during the two‐month period (S100β^+^BrdU^+^cells) was found elevated in male SAMP8 mice by almost 4‐fold compared to male SAMR1 but they remained unaltered in female SAMP8 mice compared to SAMR1. This effect was prevented in males by the two‐month ER272 treatment (Figure 5A,C). Interestingly, the number of S100β^+^BrdU^+^cells in male SAMP8 mice was around 5‐fold higher than in female SAMP8, indicating that astrogliogenesis is highly elevated in SAMP8 male mice whereas this increment was not observed in females. The study of the percentage of all BrdU^+^ cells that differentiated into astrocytes (S100β^+^ BrdU^+^ cells/total BrdU^+^ cells) showed a tendency of BrdU^+^ cells in both male and female SAMP8 mice to differentiate into S100β^+^ cells (Figure 5A,D), a tendency that was prevented by the treatment in both male and female mice (Figure 5A,D).

**FIGURE 5:**
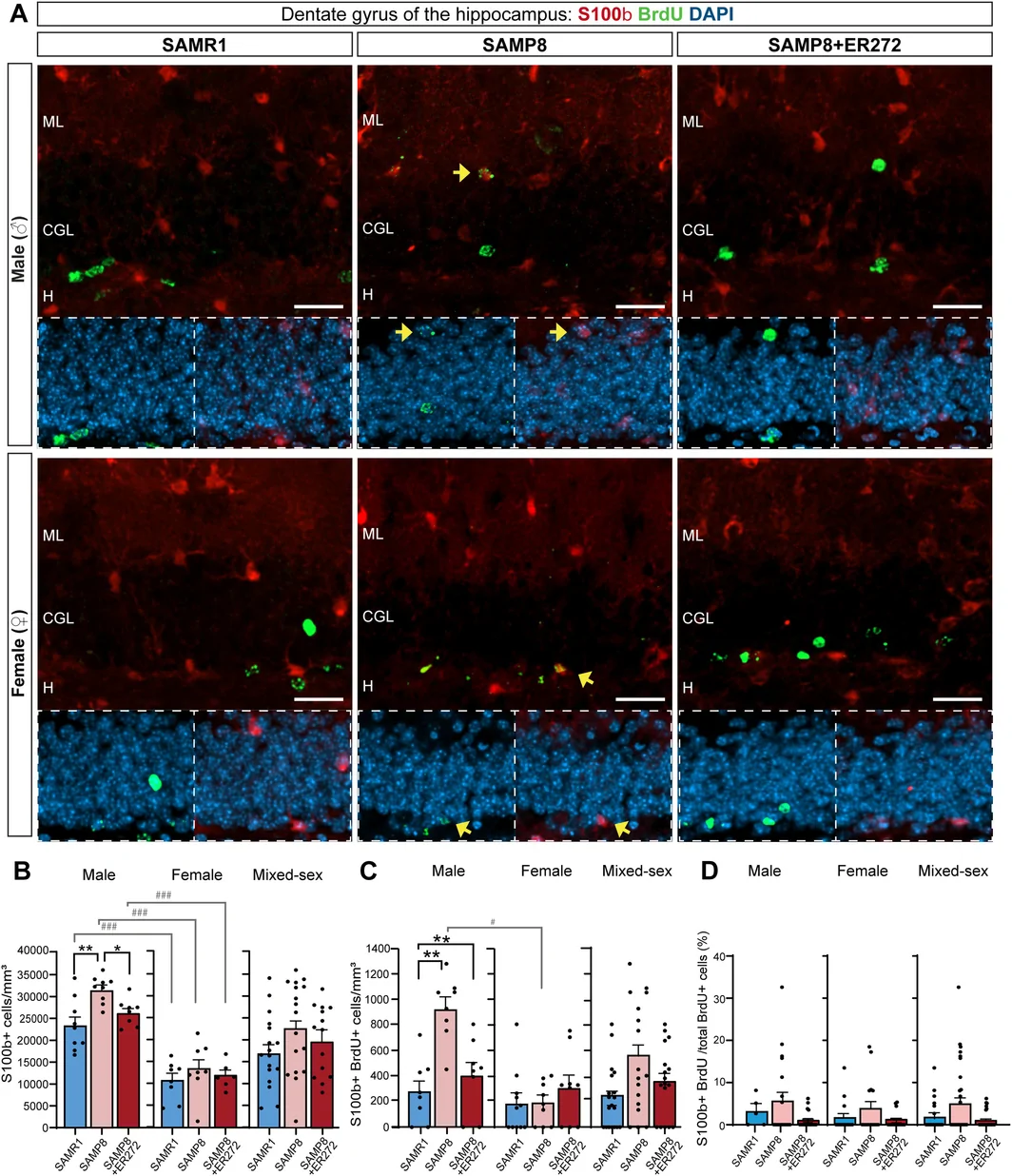
Sex differences in newly generated astrocytes in the DG of SAMP8 mice. (A) Representative confocal microscopy images of the DG of the hippocampus of six‐month‐old SAMR1 and SAMP8 male mice treated with vehicle or SAMP8 mice treated with ER272 (SAMP8 + ER272) for 8 weeks as indicated in Figure 1A. Slices were processed for the immunohistochemical detection of the proliferation marker BrdU (green), the Glial marker of mature astrocytes S100β (red) and DAPI. Yellow arrows indicate S100β^+^BrdU^+^ cells. (B) Graph shows the number of S100β^+^ cells in the DG per mm^3^ in males (left panel) [*F*(2,23) = 8.553, **p = 0.0013 SAMR1 vs. SAMP8] [*F*(2,23) = 8.553, *p = 0.047 SAMP8 vs. SAMP8 + ER272], females (center panel) [*F*(2,19) = 0.602, *p* = 0.558; no significant differences among groups], and both cohorts together (right panel) [*F*(2,45) = 1.649, *p* = 0.204; no significant differences among groups]. Sex differences in the number of S100β^+^ cells in the DG per mm^3^ are also shown for the SAMR1 group [*F*(5,42) = 29.89, ###*p* < 0.0001], the SAMP8 group [*F*(5,42) = 29.89, ###*p* < 0.0001], and the SAMP8 + ER272 group [*F*(5,42) = 29.89, ###*p* < 0.0001]. Sample size: SAMR1 (♂ *n* = 9, ♀ *n* = 8), SAMP8 (♂ *n* = 9, ♀ *n* = 8), SAMP8 + ER272 (♂ *n* = 8, ♀ *n* = 6). (C) Graph shows the number of S100β^+^BrdU^+^ cells in the DG of the hippocampus per mm^3^ in males (left panel) [*F*(2,21) = 13.83, **p = 0.002 SAMR1 vs. SAMP8] [*F*(2,21) = 13.83, **p = 0.002 SAMP8 vs. SAMP8 + ER272], females (center panel) [*p* = 0.459] and both cohorts together (right panel) [*p* = 0.06], with no significant differences detected among groups. Sex differences are also shown for the SAMP8 group [*F*(5,47) = 10.63, #*p* = 0.02]. Sample size: SAMR1 (♂ *n* = 8, ♀ *n* = 11), SAMP8 (♂ *n* = 8, ♀ *n* = 9), SAMP8 + ER272 (♂ *n* = 8, ♀ *n* = 9). (D) No statistically significant differences were detected in the percentage of S100β^+^BrdU^+^ cells relative to total BrdU^+^ cells among groups. Scale bar represents 25 μm.

### 3.5 Sex‐Specific Activation of Niche NSCs in Aged SAMP8 Mice

The pool of NSCs and their activation was then analyzed. To this end, we studied the number of cells with radial glial‐like morphology (r) expressing GFAP (rGFAP^+^) and SOX2 (rGFAP^+^SOX2^+^). 6‐month‐old male and female SAMP8 mice showed a reduction in the number of rGFAP^+^SOX2^+^ cells. The treatment induced a further reduction in the number of these cells only in males, but not in females (Figure 6A,B). The analysis of the results including mice of both sexes rendered results similar to those found in males. To assess activation of NSC, we analyzed MCM2 expression in GFAP^+^SOX2^+^ cells with radial glial‐like morphology. MCM2 is part of the DNA replication licensing complex and is expressed in cells that have entered the cell cycle, making it a useful marker of proliferating or cell‐cycle‐competent NSCs. Thus, GFAP^+^SOX2^+^MCM2^+^ triple labeling together with radial glial‐like morphology was used to distinguish activated neural stem cells from the broader pool of rGFAP^+^SOX2^+^ cells. Interestingly, males of all groups showed a higher number of activated NSCs (rGFAP^+^SOX2^+^MCM2^+^) compared to females, although this difference was not statistically significant (Figure 6A,C). However, no alterations in the number of rGFAP^+^SOX2^+^MCM2^+^ were found in SAMP8 mice compared to SAMR1, in either males or females. Furthermore, no statistically significant effect of the treatment on rGFAP^+^SOX2^+^MCM2^+^ cells was observed (Figure 6A,C) in any of the groups. Nevertheless, the percentage of activated NSCs relative to the total NSC population was 2‐fold higher in male SAMP8 mice compared to male SAMR1. This percentage increased up to 3‐fold in male SAMP8 mice as a consequence of the treatment (Figure 6A,D). In contrast, no changes in the percentage of activated NSCs were observed in female SAMP8 mice compared to SAMR1 (Figure 6A,D) and the treatment did not exert any effect on this percentage. The analysis using animals of both sexes rendered results similar to those found in male mice (Figure 6), emphasizing the importance of analyzing sexes separately in this type of studies.

**FIGURE 6:**
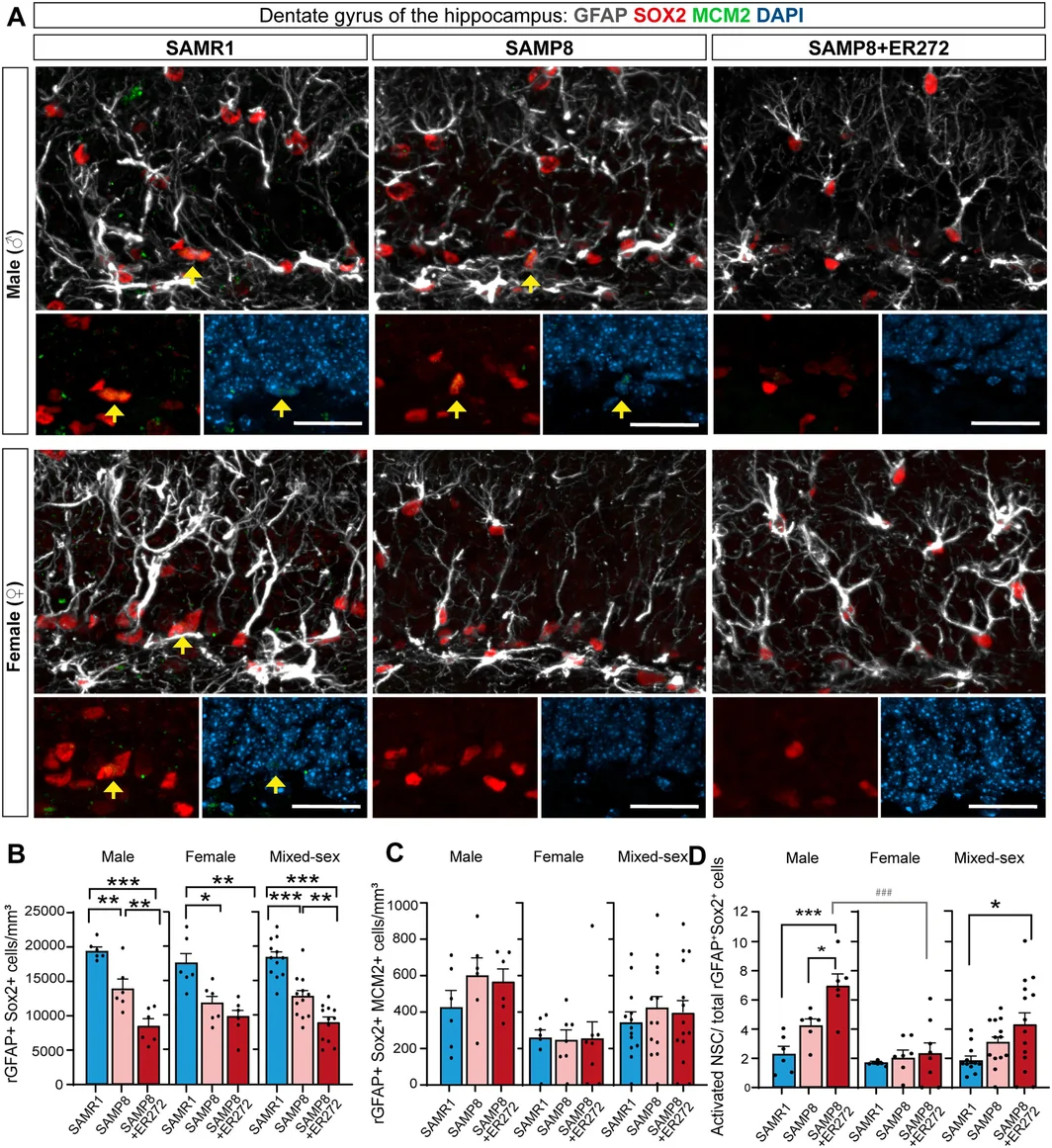
Sex dependent activation of NSC in the DG of SAMP8 mice and response to treatment. (A) Representative confocal microscopy images of the DG of the hippocampus of six‐month‐old SAMR1 and SAMP8 male mice treated with vehicle or SAMP8 mice treated with ER272 during 8 weeks as indicated in Figure 1A. Tissue processed for the detection of the Glial Fibrillary Acidic Protein GFAP (white), SOX2 (red) and the cell cycle marker MCM2 (green). Yellow arrows indicate rGFAP^+^MCM2^+^SOX2^+^ cells. (B) Graph shows the total number of rGFAP^+^SOX2^+^ cells with a radial glial‐like morphology in the DG per mm^3^ in males (left panel) [*F*(2,15) = 27.92, **p = 0.004 SAMR1 vs. SAMP8] [*F*(2,15) = 27.92, ***p < 0.001 SAMR1 vs. SAMP8 + ER272] [*F*(2,15) = 27.92, **p = 0.004 SAMP8 vs. SAMP8 + ER272], females (center panel) [*F*(2,15) = 10.30, *p = 0.018 SAMR1 vs. SAMP8] [*F*(2,15) = 10.30, **p = 0.009 SAMR1 vs. SAMP8 + ER272], and both cohorts together (right panel) [*F*(2,33) = 33.03, ***p < 0.001 SAMR1 vs. SAMP8] [*F*(2,33) = 33.03, ***p < 0.001 SAMR1 vs. SAMP8 + ER272] [*F*(2,33) = 33.03, **p = 0.0043 SAMP8 vs. SAMP8 + ER272]. No sex differences were detected in the total number of rGFAP^+^SOX2^+^ cells. Sample size: SAMR1 (♂ *n* = 6, ♀ *n* = 6), SAMP8 (♂ *n* = 6, ♀ *n* = 6), SAMP8 + ER272 (♂ *n* = 6, ♀ *n* = 6). (C) Graph shows the number of rGFAP^+^SOX2^+^MCM2^+^ cells with a radial glial‐like morphology in the DG per mm^3^ in males (left panel) [*F*(2,15) = 1.136, *p* = 0.347], females (center panel) [*F*(2,19) = 0.0068, *p* = 0.993], and both cohorts together (right panel) [*F*(2,37) = 0.305, *p* = 0.739]. No significant differences were detected among groups or between sexes. Sample size: SAMR1 (♂ *n* = 6, ♀ *n* = 7), SAMP8 (♂ *n* = 6, ♀ *n* = 7), SAMP8 + ER272 (♂ *n* = 6, ♀ *n* = 8). (D) Percentage of activated NSCs (rGFAP^+^SOX2^+^MCM2^+^) among the total NSC population (rGFAP^+^SOX2^+^) in the DG of males (left panel) [*F*(2,15) = 14.26, ***p < 0.001 SAMR1 vs. SAMP8 + ER272] [*F*(2,15) = 14.26, *p = 0.019 SAMP8 vs. SAMP8 + ER272], females (center panel) [*F*(2,18) = 0.344, *p* = 0.714; no significant differences among groups], and both cohorts together (right panel) [*F*(2,36) = 3.982, *p = 0.021 SAMR1 vs. SAMP8 + ER272]. Sample size: SAMR1 (♂ *n* = 6, ♀ *n* = 6), SAMP8 (♂ *n* = 6, ♀ *n* = 7), SAMP8 + ER272 (♂ *n* = 6, ♀ *n* = 8). Sex differences in the percentage of activated NSCs are also shown for the SAMP8 + ER272 group [*F*(5,33) = 10.71, ###*p* < 0.001]. Scale bar represents 25 μm.

### 3.6 Analysis of Combined Cohorts Show a Clear Bias Toward the Male Phenotype

Across the different parameters analyzed in this study (newly generated neurons, mature neurons, astroglial cells, NSCs, and cognitive performance), pooling data from both sexes resulted in values that were largely similar to those found in male mice. With the exception of the total number of NSCs, mixed‐sex analyses failed to capture the female‐specific patterns observed when sexes were analyzed separately.

## 4 Discussion

Cognitive performance tends to deteriorate with age, and aging itself constitutes a major independent risk factor for neurodegenerative disorders affecting both sexes (Anstey et al. 2021; Cicali et al. 2025). Certain memory domains, particularly episodic memory, appear to be especially vulnerable in older individuals (Gray and Barnes 2015; Nyberg et al. 2012). As a result, identifying effective strategies to prevent or mitigate age‐related cognitive decline has become a priority. Among the potential therapeutic approaches, potentiating adult hippocampal neurogenesis is a promising strategy to reduce cognitive deterioration in aging populations. However, to accelerate therapeutic discovery, it is crucial to examine sex differences. Experimental studies show that spatial training paradigms such as the MWM or pattern separation tasks selectively enhance DG neurogenesis in male but not female rats (Chow et al. 2013; Yagi and Galea 2019). These sex‐dependent effects on neurogenesis align with a superior behavioral outcome generally observed in males, raising the possibility that neurogenic responses may underlie at least part of the differences in cognitive performance found between sexes. We have previously identified molecules that enhance neurogenesis in the adult brain and proposed their potential as pharmacological interventions for cognitive deficits associated with impaired neurogenesis. Among these, the diterpene ER272, characterized by a 12‐deoxyphorbol structure (Geribaldi‐Doldan et al. 2015; Gomez‐Oliva et al. 2023), was administered to male SAMP8 mice prior to the onset of cognitive decline, effectively preventing cognitive impairment. In the present study, we investigated whether the neurogenic and cognitive benefits previously described in male SAMP8 mice could be extrapolated to females. In doing so, we unearthed marked sex‐dependent differences in neurogenic profiles and treatment responsiveness, suggesting that males and females may follow distinct age‐dependent neurogenic adaptations.

### 4.1 Prevention of Cognitive Decline by Diterpene ER272 in Male Versus Female

The behavioral assessment, using the MWM and NOD test, revealed that both male and female show a reduction in the capacity to perform these tasks at 6 months of age. However, only males benefited from the ER272 treatment, which prevented the decline in cognitive performance. Our results are consistent with previous findings reporting cognitive deficits in 6‐month‐old SAMP8 male mice (Gomez‐Oliva et al. 2023; Pačesová et al. 2022). Importantly, our findings provide novel insights into female SAMP8 mice and suggest that despite exhibiting similar cognitive performance at 6 months of age, males and females respond differently to therapeutic intervention. These differences may reflect underlying sex‐specific mechanisms governing vulnerability or resilience to age‐related neurodegeneration. Several reports have shown a clear male advantage in spatial memory using different tests (Hausmann et al. 2000; LaBuda et al. 2002; Rodríguez et al. 2011). Similarly, other reports that use aged male and female C57BL/6J as well as 10‐month‐old SAMP8 mice reveal sex‐dependent differences in cognitive impairment, with aged males exhibiting more significant deficits in spatial memory, while aged females show impairments in recognition memory (Cicali et al. 2025). In contrast, we detected no sex‐specific differences in performance in the NOD and MWM between control and 6‐month‐old SAMP8 mice, indicating that neither sex displayed a clear behavioral advantage at this stage.

A possible explanation is that 6 months of age represents a transitional stage at which both male and female SAMP8 mice exhibit a comparable degree of cognitive impairment. Thus, sex‐dependent behavioral differences reported in other studies (Cicali et al. 2025) may emerge either at later stages of aging, when pathological processes have diverged more markedly between sexes. In support of this interpretation, ER272 improved cognitive performance exclusively in males, suggesting that sex‐specific neurobiological mechanisms may already be operating despite the absence of clear behavioral differences between sexes at this age.

### 4.2 Sex‐Dependent Differences in Dentate Gyrus Neurogenesis Despite Similar Cognitive Performance

Sex differences have been reported in pattern separation, pattern completion, spatial learning, and their links to adult neurogenesis, highlighting the need to include both sexes when studying hippocampal cellular and structural mechanisms (Yagi and Galea 2019). Consistent with previous reports, male SAMR1 mice in our study exhibited higher levels of newly generated immature neurons than age‐matched females, supporting the existence of intrinsic sex‐dependent differences in baseline hippocampal neurogenesis and its regulation by hormonal, inflammatory, and aging‐related mechanisms. A decline in hippocampal neurogenesis due to aging and neurodegeneration has been previously reported not only in murine models like the SAMP8 mice (Diaz‐Moreno et al. 2018; Diaz‐Moreno et al. 2013) or AD genetic models (Choi et al. 2018; Zaletel et al. 2018) but also in humans (Marquez‐Valadez et al. 2022; Terreros‐Roncal et al. 2021). Recent single‐cell multiomic analyses of human hippocampi have demonstrated that aging and Alzheimer's disease are associated with early alterations in NSC, neuroblasts, and immature neurons, supporting the idea that disruption of neurogenic processes is a conserved feature of brain aging across species (Disouky et al. 2026). However, our findings suggest that this decline does not occur identically in male and female SAMP8 mice. We have analyzed the sex‐specific difference in neurogenesis of 6‐month‐old SAMP8 mice. Remarkably, males exhibited pronounced alterations in immature neuron generation, neuronal maturation, astrogliogenesis, and NSC activation, whereas females showed comparatively limited changes. These observations support the notion that males and females may follow distinct neurogenic trajectories during the aging process rather than representing different degrees of the same pathological process. Interestingly, the beneficial effects of ER272 were largely restricted to males where treatment prevented cognitive decline and restored several neurogenic parameters. These results were consistent with previous findings (Gomez‐Oliva et al. 2023). In contrast, females displayed little responsiveness to the treatment. One possible explanation is that the lower baseline neurogenic activity observed in females may reduce the dynamic range available for neurogenic modulation. Alternatively, males and females may be at different stages of pathological progression at 6 months of age, resulting in distinct treatment responsiveness. Although the number of newly generated mature neurons was not significantly altered by aging, males consistently exhibited higher values than females across experimental groups. Together with the increased activation of NSC observed in males, these findings suggest that ER272 may primarily act by expanding the proliferative pool of progenitors rather than by directly enhancing neuronal differentiation. Nevertheless, because BrdU was administered repeatedly throughout the treatment period to label cells generated during ER272 exposure, the present design reflects the cumulative neurogenic outcome of the treatment and does not allow discrimination between effects on proliferation, survival, and lineage progression. Future studies using pulse‐chase paradigms will be required to determine the specific neurogenic stages targeted by ER272.

### 4.3 Sex‐Dependent Differences in Neuronal Maturation and Morphological Development

Previous works using male SAMP8 mice show dysfunction and aberrant maturation of immature neurons that may correlate with our present findings (Diaz‐Moreno et al. 2018; Diaz‐Moreno et al. 2013). Consistent with these observations, we found marked alterations in the morphology of immature neurons in male SAMP8 mice, whereas female SAMP8 mice showed no evidence of impaired neuronal maturation. These findings further support the notion that males and females follow distinct neurogenic aging trajectories. Interestingly, the morphological abnormalities observed in male SAMP8 mice were not accompanied by a significant reduction in the total number of newly generated mature neurons. This suggests that the neurogenic impairment associated with aging may primarily affect early stages of neuronal differentiation and maturation rather than the final survival of newborn neurons. Consequently, a proportion of newly generated cells may still acquire a mature neuronal phenotype despite exhibiting abnormal developmental progression. ER272 treatment prevented the morphological alterations observed in male SAMP8 mice, indicating that its beneficial effects extend beyond the regulation of NSC activation and cell fate decisions. Together with the reduction in astroglial commitment, these findings suggest that ER272 promotes the progression of newborn neurons through immature stages and facilitates their structural maturation in male as previously reported (Gomez‐Oliva et al. 2023). Although these findings support a role for ER272 in the modulation of hippocampal neurogenesis, we did not directly assess its biodistribution following intranasal administration. Therefore, we cannot conclusively demonstrate that ER272 reaches the hippocampus. However, previous studies using the same administration route have demonstrated beneficial effects on cognitive performance and modulation of cerebrospinal fluid biomarkers, supporting the existence of central biological effects (Dominguez‐Garcia et al. 2021; Gomez‐Oliva et al. 2023). Future pharmacokinetic and biodistribution studies will be necessary to determine the extent of ER272 delivery to specific brain regions and to clarify whether the neurogenic effects reported here result from direct hippocampal actions or indirect modulation of central signaling pathways.

### 4.4 Age‐Dependent Astrogliogenesis in the DG Can Be Prevented in Male but Not in Female With Diterpene Treatment

Previous reports have shown that hippocampal aging is characterized by the differentiation of NSCs into astroglial cells leading to the loss of NSC in the DG of aged mice due to their preferential differentiation into mature astrocytes (Diaz‐Moreno et al. 2018; Encinas et al. 2011). Consistent with these observations, we found an increased number of newly generated astrocytes in male SAMP8 mice compared with SAMR1 controls, suggesting that astrogliogenesis is enhanced during this stage of pathological aging. Notably, ER272 treatment prevented this increase, indicating that the compound can counteract the age‐related shift toward astroglial differentiation. In contrast, female SAMP8 mice did not exhibit increased astrogliogenesis relative to SAMR1 controls, and consequently no effect of ER272 was observed. These findings further support the idea that males and females follow distinct neurogenic aging trajectories and may be at different stages of disease progression at 6 months of age. Interestingly, although total astrocyte numbers increased with aging in both sexes, females consistently displayed lower astrocyte numbers than males. Together with the reduction in immature neurons and the increased activation of NSCs observed in male SAMP8 mice, these findings suggest that activated NSCs may preferentially adopt an astroglial rather than a neuronal fate. This interpretation is further supported by the increased number of newly generated astrocytes observed in male SAMP8 mice. Consistent with previous reports (Diaz‐Moreno et al. 2018), these findings suggest astrogliogenesis is favored in male SAMP8 mice, and that ER272 is able to prevent this shift in cell fate commitment.

### 4.5 Sex‐Dependent Activation of NSCs

Evidence shows that the proportion of NSC decreases with age in the mouse brain. Concomitantly, a reduced ability of NSC to undergo activation is found that allows them to remain quiescent for prolonged periods of time avoiding exhaustion of the NSC pool (Diaz‐Moreno et al. 2018; Harris et al. 2021; Ibrayeva et al. 2021; Martin‐Suarez et al. 2019). Consistent with previous reports (Diaz‐Moreno et al. 2018) both male and female SAMP8 mice exhibited a reduced NSC pool compared with SAMR1 controls, as reflected by the lower number of SOX2^+^GFAP^+^ radial glia‐like cells. Despite this reduction, male SAMP8 mice showed an increased proportion of activated NSCs, which was further enhanced by ER272 treatment. In contrast, no changes in NSC activation were observed in females. Previous studies have identified two distinct neural stem cell populations in the aged mouse brain: one with high activation and neurogenic potential, and another characterized by reduced proliferative activity and limited contribution to neurogenesis (Harris et al. 2021; Ibrayeva et al. 2021; Martin‐Suarez et al. 2019). The differential response observed between sexes may therefore reflect differences in the relative abundance of these NSC populations. In particular, males may retain a larger pool of resting, non‐dormant NSCs capable of re‐entering the cell cycle in response to treatment, thereby contributing to the greater neurogenic plasticity observed in the aged male hippocampus.

### 4.6 Neurogenesis in Female SAMP8 Mice Versus Male

In contrast to males, female SAMP8 mice did not exhibit reductions in the number and morphological abnormalities of newly generated immature neurons. Consistently with the absence of alterations, ER272 treatment failed to significantly modify these parameters. Female SAMP8 mice also lacked the increase in activated neural stem cells observed in males, despite showing a reduction in the total NSC pool. Together, these findings suggest that the neurogenic alterations observed in male SAMP8 mice are either absent or substantially attenuated in females. Interestingly, despite the absence of clear alterations in early neurogenic stages, the total number of newly generated mature neurons was markedly lower in females than in males across all experimental groups. This finding suggests the existence of intrinsic sex‐dependent differences in the generation, maturation, or long‐term survival of newborn neurons in the DG. A plausible explanation is that male and female SAMP8 mice follow distinct neurogenic divergent patterns of neurogenic aging decline. Whereas males develop pronounced impairments affecting early neuronal differentiation and astroglial commitment, females may exhibit a lower basal neurogenic capacity without manifesting the same pathological alterations detected in males at this age. Consequently, the limited responsiveness to ER272 in females may reflect the absence of detectable neurogenic deficits rather than a complete lack of treatment sensitivity.

### 4.7 Possible Influence of Sex Hormones on the Observed Effects

Sex differences in hippocampal function and adult neurogenesis are known to be influenced, at least in part, by ovarian hormones. Previous studies have shown that fluctuations in ovarian hormones across the estrous cycle can influence adult hippocampal neurogenesis, neuronal survival, and hippocampus‐dependent behaviors, particularly during phases associated with elevated estrogen levels (Galea et al. 2006; Yagi and Galea 2019). Therefore, estrous cycle‐dependent hormonal variations may have contributed to the variability observed in female animals in the present study. Although estrous cyclicity was not monitored in the present study, the duration of the treatment (2 months) involved multiple estrous cycles, reducing the likelihood that our findings are driven by transient cycle‐dependent effects. Moreover, the consistent and directional nature of the sex‐specific differences observed in behavior and neurogenesis suggests that they reflect stable biological differences between males and females rather than fluctuations associated with cycle stage. Nevertheless, we cannot exclude that hormonal status may modulate the magnitude of these effects, and future studies incorporating estrous cycle monitoring or hormonal profiling will be important to further dissect the underlying mechanisms. Sex hormones may contribute to the observed sex‐dependent differences in hippocampal neurogenesis and treatment responsiveness. Estrogens are known to modulate neural stem cell proliferation, neuronal differentiation, and survival in the adult hippocampus, and may also exert neuroprotective and anti‐inflammatory effects (Bustamante‐Barrientos et al. 2021; Galea et al. 2006; Yagi et al. 2020). Therefore, hormonal differences between males and females could partially explain the distinct neurogenic profiles observed in SAMP8 mice. However, since hormone levels were not evaluated in the present study, this possibility remains speculative and should be addressed in future investigations.

### 4.8 Sex Bias in Mixed Cohorts: Male‐Driven Outcomes in Aging Studies

In most of our studies using mixed‐sex cohorts of SAMP8 mice, the resulting phenotype closely resembled that of males, rather than representing an intermediate or averaged response. This male bias in mixed analyses has been previously described in multiple preclinical studies, where male data often dominate due to greater variability or a stronger expression of age‐related phenotypes. In agreement with previous reports, these findings reinforce the importance of considering sex as a biological variable in experimental design and data interpretation, in order to uncover mechanisms that may be unique to the female brain (Beery and Zucker 2011; Shansky and Woolley 2016).

## 5 Conclusion

In conclusion, our findings reveal marked sex‐dependent differences in hippocampal neurogenesis during physiological and pathological aging in SAMP8 mice. Despite exhibiting similar cognitive performance at 6 months of age, male and female mice displayed distinct neurogenic profiles and responded differently to ER272 treatment. Males showed alterations in NSC activation, cell fate commitment, and neuronal maturation that were largely restored by the treatment, whereas females exhibited a comparatively preserved neurogenic profile and limited treatment responsiveness. These observations suggest that males and females may not simply differ in the severity of neurogenic decline but rather follow distinct neurogenic aging trajectories. By challenging the notion of a uniform aging process, our study highlights the importance of considering sex as a biological variable in studies of brain aging and supports the development of sex‐informed therapeutic strategies targeting neurogenic plasticity.

## Author Contributions

**Ricardo Gómez‐Oliva, Pedro Nunez‐Abades, Mónica García‐Alloza:** experimental design, data acquisition and analysis, discussion of results article preparation and writing. **Isabel Atienza‐Navarro, Andrea Chamorro‐Francisco, Livia Carrascal, María D. Freire‐Aragón:** data acquisition and analysis, discussion of results, preparation and writing. **Rosario Hernández‐Galán:** isolation characterization and preparation of chemical compound. **Carmen Castro:** conception of the work, experimental design, data acquisition and analysis, discussion of results, article preparation and writing, funding acquisition.

## Funding

This publication is part of the I+D+i (PID2022‐142418OB‐C21 and PID2022‐142418OB‐C22) grant funded by MICIU/AEI/10.13039/501100011033 and by ERDF/UE. This work was also supported by the University of Cadiz through the 2022–2023 Internal Plan for Support and Promotion of Research and Knowledge Transfer, co‐funded by the Andalusia 2021–2027 ERDF/UE Operational Program (FEDER‐UCA‐2024‐A2‐13).

## Conflicts of Interest

The authors declare no conflicts of interest.

## Supporting information


**Table S1:** Primary antibodies. List of primary antibodies used in the study. Specifying host, isotype, epitope retrieval, staining pattern, source and reference.
**Table S2:** Secondary antibodies. List of secondary antibodies used in the study. Specifying host, dilution used, fluorescence conjugated, source and reference.
**Table S3:** Statistical comparisons for the acquisition phase of the Morris water maze test.
**Table S4:** Time spent in each of the four quadrants in the 24h retention phase of the MWM (males and females).
**Table S5:** Summary of data and significant sex‐related differences (*p* values) in neurogenic parameters from statistical comparisons between female and male animals, using one‐way ANOVA with Tukey's post hoc test (parametric) or Kruskal–Wallis with Dunn's test (non‐parametric).

## Data Availability

All datasets generated and/or analyzed during the current study will available from the corresponding author upon reasonable request.
