# Supplementary material for: Sex‐Specific Neurogenic and Cognitive Responses in a Murine Model of Accelerated Aging
Source: Aging Cell. 2026 Oct 9;25(10):e70663. doi: 10.1111/acel.70663 (PMC13651790; doi:10.1111/acel.70663)
Supplement: Supplementary file 1 [file ACEL-25-e70663-s001.docx]

**Supplementary table 1. Primary antibodies.** List of primary antibodies used in the study. Specifying host, isotype, epitope retrieval, staining pattern, source and reference.

| Antibody | Host | Isotype | Epitope retrieval | Staining pattern | Source | Reference |
| --- | --- | --- | --- | --- | --- | --- |
| Anti-DCX | Rabbit | Polyclonal | DCX, neuroblast marker | Cytoplasmic | Abcam (Cambridge, UK) | ab18723 |
| Anti-S100β | Rabbit | Polyclonal | S100β, astrocyte  marker | Cytoplasmic | Abcam (Cambridge, UK) | ab41548 |
| Anti-NeuN | Rabbit | Monoclonal | NeuN, neuronal marker | Nuclear | Abcam (Cambridge, UK) | ab177487 |
| Anti-SOX2 | Rabbit | Polyclonal | SOX2,  astrocyte/stem cell marker | Nuclear | Santa Cruz Biotechnology, Santa Cruz, CA, USA) | sc-20088 |
| Anti-SOX2 | Goat | Polyclonal |  | Nuclear | RD Systems Minneapolis, CO, USA) | AF2018 |
| Anti-MCM2 | Mouse | Monoclonal | MCM2, cell proliferation marker | Nuclear | BD Bioscience  ( East Rutherford, NJ, USA) | 610701 |
| Anti-GFAP | Chicken | Polyclonal | GFAP, glial marker | Cytoplasmic | Abcam (Cambridge, UK) | ab4674 |
| Anti-NeuN | Rat | Monoclonal | NeuN, neuronal marker | Nuclear | Abcam (Cambridge, UK) | ab279297 |
| Anti-BrdU | Rat | Monoclonal | BrdU, cell proliferation marker | Nuclear | Abcam (Cambridge, UK) | ab6362 |

**Supplementary table 2: Secondary antibodies.** List of secondary antibodies used in the study. Specifying host, dilution used, fluorescence conjugated, source and reference

| Antibody | Host | Dilution | Fluorescence | Source | Reference |
| --- | --- | --- | --- | --- | --- |
| Alexa Flour anti-rabbit | Donkey | 1:1000 | 647 | Invitrogen (Carlsbad, CA, USA) | A-32795 |
| Alexa Flour anti-rabbit | Donkey | 1:1000 | 594 | Invitrogen (Carlsbad, CA, USA) | A-21207 |
| Alexa Flour anti-rabbit | Donkey | 1:1000 | 488 | Invitrogen (Carlsbad, CA, USA) | A-21206 |
| Alexa Flour anti-goat | Donkey | 1:1000 | 647 | Invitrogen (Carlsbad, CA, USA) | A-11058 |
| Alexa Flour anti-mouse | Donkey | 1:1000 | 488 | Invitrogen (Carlsbad, CA, USA) | A-21206 |
| Alexa Flour anti-chicken | Goat | 1:1000 | 647 | Abcam (Cambridge, UK) | ab150175 |
| Alexa Flour anti-rat | Donkey | 1:1000 | 488 | Life Technologies (Eugene, OR, USA) | A-21208 |

.

**Supplementary table 3. Statistical comparisons for the acquisition phase of the Morris water maze test.**

| Group | 2-way ANOVA (dayXsex) | Day effect | Sex effect |
| --- | --- | --- | --- |
| **SAMR1** | [F_(3,306)_=0.138, p=0.937] | F=20.55 p<0.001 | F=37.82; p<0.001 |
| **SAMP8** | [F_(3,314)_=0.257, p=0.857] | F=16.61; p<0.001 | F=7.10; p=0.008 |
| **SAMP8-ER** | [F_(3,337)_=0.957, p=0.413] | F=7.69; p<0.001 | F=2.49; p<0.001 |

**Supplementary table 4. Time spent in each of the four quadrants in the 24h retention phase of the MWM (males and females)**

|  | Retention 24 h (%time) | | | | | | | |
| --- | --- | --- | --- | --- | --- | --- | --- | --- |
|  | **Males** | | | | **Females** | | | |
| **Group** | **Quadrant**  **1** | **Quadrant**  **2** | **Quadrant**  **3** | **Quadrant**  **4** | **Quadrant**  **1** | **Quadrant**  **2** | **Quadrant**  **3** | **Quadrant**  **4** |
| **SAMR1** | 23.13±3,19 | 46.30±4.40 | 15.68±2.91 | 14.60±3.60 | 21.77±3.29 | 37.50±5.51 | 25.82±5.45 | 14.64±3.10 |
| **SAMP8** | 25.88±3,48 | 28.82±3.32‡ | 21.20±3.15 | 23.89±3.28 | 22.46±3.81 | 32.38±4.87 | 20.50±2.52 | 24.39±3.57 |
| **SAMP8-ER** | 17.58±2,79 | 42.25±4.70 | 24.15±5.72 | 15.76±3.21 | 31.07±3,52 | 35.26±3.87 | 17.62±2.15 | 15.77±2.53 |

Male SAMP8 mice spent significantly shorter times in quadrant 2, where the platform used to be located [F(2,25)=4.16, ‡p=0.028 vs. SAMR1]. No differences were detected between groups when we compared the time males spent in quadrant 1 [F(2,25)=1.78, p=0.188], quadrant 3 [F(2,25)=1.078, p=0.356] or quadrant 4 [F(2,25)=2.03, p=0.152]. No differences were observed when time females spent in quadrant 1 [F(2,29)=2.12, p=0.137], quadrant 2 [F(2,29)=0.288, p=0.752], quadrant 3 [F(2,29)=1.33, p=0.278] or quadrant 4 [F(2,29)=2.98, p=0.066].

**Table 5. Summary of data and significant sex-related differences (*p* values) in neurogenic parameters from statistical comparisons between female and male animals, using one-way ANOVA with Tukey’s post hoc test (parametric) or Kruskal–Wallis with Dunn’s test (non-parametric).**

|  | **Conditions** | | | |  |
| --- | --- | --- | --- | --- | --- |
| **Parameters** | **SAMR1** | **SAMP8** | **SAMP8+ER272** | **Statistically significant sex differences** | |
| **BrdU^+^ cells/mm^3^** |  |  |  |  | |
| Male | 8,769 ± 1087 (n=10) | 6,637 ± 934  (n=12) | 12,417 ± 953  (n=12) | ♂ R1 vs ♂ P8: p value = 0.30 ♂ R1 vs ♂ P8+ER: p value = 0.02 ♂ P8 vs ♂ P8+ER: p value = 0.0002 ♀ R1 vs ♀ P8: p value = 0.26 ♀ R1 vs ♀ P8+ER: p value = 0.46 ♀ P8 vs ♀ P8+ER: p value = 0.03 ♂ R1 vs ♀ R1: p value = 0.33 ♂ P8 vs ♀ P8: p value = 0.35 ♂ P8+ER vs ♀ P8+ER: p value = 0.005 Mixed R1 vs. Mixed P8 p value =0.18; Mixed R1 vs. MIxed P8 +ER p value =0.01; Mixed P8 vs. MIxed P8 +ER p value <0.0001 | |
| Female | 5,927 ± 845  (n=9) | 4,045 ± 901  (n=10) | 7,465 ± 742  (n=7) |  |  |
| Mixed-gender | 7,423± 757  (n=19) | 5,459± 698  (n=22) | 10,877± 892  (n=19) |  |  |
| **DCX^+^ BrdU+ cells/mm3** |  |  |  |  | |
| Male | 5,401 ± 1115 (n=9) | 1988 ± 517 (n=12) | 5,922 ± 832 (n=12) | ♂ R1 vs ♂ P8: p value = 0.02 ♂ R1 vs ♂ P8+ER: p value =0.89 ♂ P8 vs ♂ P8+ER: p value = 0.003 ♀ R1 vs ♀ P8: p value >0.99 ♀ R1 vs ♀ P8+ER: p value =0.43 ♀ P8 vs ♀ P8+ER: p value =0.37 ♂ R1 vs ♀ R1: p value = 0.016 ♂ P8 vs ♀ P8: p value >0.99 ♂ P8+ER vs ♀ P8+ER: p value = 0.17 Mixed R1 vs. Mixed P8 p value =0.04; Mixed R1 vs. MIxed P8 +ER p value =0.07; Mixed P8 vs. MIxed P8 +ER p value<0.0001 | |
| Female | 1,278 ± 522 (n=10) | 1041 ± 293 (n=11) | 1,970 ± 389  (n=7) |  |  |
| Mixed-gender | 3,340 ± 778  (n=19) | 1,535.2 ± 314  (n=23) | 4465 ± 699 (n=19) |  |  |
| **NeuN^+^ BrdU^+^ cells/mm3** |  |  |  |  | |
| Male | 4,557 ± 299 (n=6) | 3,952 ± 117  (n=6) | 6,722 ± 463  (n=6) | ♂ R1 vs ♂ P8: p value=0.52 ♂ R1 vs ♂ P8+ER: p value=0.09 ♂ P8 vs ♂ P8+ER: p value=0.0013 ♀ R1 vs ♀ P8: p value =0.34 ♀ R1 vs ♀ P8+ER: p value =0.49 ♀ P8 vs ♀ P8+ER: p value=0.07 ♂ R1 vs ♀ R1: p value =0.08 ♂ P8 vs ♀ P8: p value = 0.03 ♂ P8+ER vs ♀ P8+ER: p value= 0.011 Mixed R1 vs. Mixed P8 p value =>0.99 Mixed R1 vs. MIxed P8 +ER p value=0.65; Mixed P8 vs. MIxed P8 +ER p value=0.13 | |
| Female | 1,541 ± 176 (n=10) | 805 ± 48 (n=6) | 2,087 ± 258 (n=8) |  |  |
| Mixed-gender | 2,672.1 ± 406 (n=16) | 2,378.7 ± 477 (n=12) | 4,073.4 ± 734 (n=14) |  |  |
| **S100β^+^ cells/mm3** |  |  |  |  | |
| Male | 23,250 ± 1982 (n=9) | 31,492 ± 1011 (n=9) | 26,216 ± 1070 (n=8) | ♂ R1 vs ♂ P8: p value=0.0013 ♂ R1 vs ♂ P8+ER: p value=0.34 ♂ P8 vs ♂ P8+ER: p value=0.047 ♀ R1 vs ♀ P8: p value =0.53 ♀ R1 vs ♀ P8+ER: p value=0.91 ♀ P8 vs ♀ P8+ER: p value=0.82 ♂ R1 vs ♀ R1: p value<0.0001 ♂ P8 vs ♀ P8: p value <0.0001 ♂ P8+ER vs ♀ P8+ER: p value <0.0001 Mixed R1 vs. Mixed P8 p value=0.17; Mixed R1 vs. MIxed P8 +ER p value=0.68; Mixed P8 vs. MIxed P8 +ER p value=0.64 | |
| Female | 10,725 ± 1544(n=8) | 13,333 ± 2126(n=8) | 11,814 ± 1238(n=6) |  |  |
| Mixed-gender | 17,356 ±1442 (n=17) | 22946 ± 736 (n=17) | 20,044 ± 1,135 (n=14) |  |  |
| **S100β^+^ BrdU^+^ cells/mm3** |  |  |  |  | |
| Male | 280 ± 86 (n=8) | 922 ± 88 (n=8) | 401 ± 101(n=8) | ♂ R1 vs ♂ P8: p value=0.002 ♂ R1 vs ♂ P8+ER: p value=>0.99 ♂ P8 vs ♂ P8+ER: p value=0.002 ♀ R1 vs ♀ P8: p value > 0.99 ♀ R1 vs ♀ P8+ER: p value =0.82 ♀ P8 vs ♀ P8+ER: p value> 0.99 ♂ R1 vs ♀ R1: p value> 0.99 ♂ P8 vs ♀ P8: p value=0.02 ♂ P8+ER vs ♀ P8+ER: p value > 0.99 Mixed R1 vs. Mixed P8 p value=0.06; Mixed R1 vs. MIxed P8 +ER p value=0.57; Mixed P8 vs. MIxed P8 +ER p value=0.92 | |
| Female | 183 ± 88 (n=11) | 186 ± 62 (n=9) | 305 ± 100 (n=9) |  |  |
| Mixed-gender | 228.0 ± 56 (n=19) | **532.9 ± 60 (n=17)** | **350.2 ± 68**  **(n=17)** |  |  |
|  |  |  |  |  |  |
| **rGFAP^+^ Sox2^+^ cells/mm3** |  |  |  |  | |
| Male | 19,389 ± 520  (n=6) | 13,912 ± 1304 (n=6) | 8,486 ± 1123 (n=6) | ♂ R1 vs ♂ P8: p value=0.0038 ♂ R1 vs ♂ P8+ER: p value=0.001 ♂ P8 vs ♂ P8+ER: p value=0.0038 ♀ R1 vs ♀ P8: p value=0.018 ♀ R1 vs ♀ P8+ER: p value=0.0086 ♀ P8 vs ♀ P8+ER: p value=0.37 ♂ R1 vs ♀ R1: p value=0.65 ♂ P8 vs ♀ P8: p value>=0.65 ♂ P8+ER vs ♀ P8+ER: p value =0.65 Mixed R1 vs. Mixed P8 p value<0.001; Mixed R1 vs. MIxed P8 +ER p value<0.001; Mixed P8 vs. MIxed P8 +ER p value=0.0043 | |
| Female | 17,756 ± 1,514 (n=6) | 11,921 ± 1128  (n=6) | 9,959 ± 1105  (n=6) |  |  |
| Mixed-gender | 18,906 ± 727 (n=12) | 12,916 ± 856 (n=12) | 9,222.7 ± 782 (n=12) |  |  |
|  | | | |  |  |
| **rGFAP^+^ Sox2^+^ MCM2^+^ cells/mm3** | |  |  | ♂ R1 vs ♂ P8: p value=0.43 ♂ R1 vs ♂ P8+ER: p value=0.46 ♂ P8 vs ♂ P8+ER: p value=0.78 ♀ R1 vs ♀ P8: p value> 0.99 ♀ R1 vs ♀ P8+ER: p value> 0.99 ♀ P8 vs ♀ P8+ER: p value> 0.99 ♂ R1 vs ♀ R1: p value=0.72 ♂ P8 vs ♀ P8: p value=0.06 ♂ P8+ER vs ♀ P8+ER: p value =0.106 Mixed R1 vs. Mixed P8 p value=0.73; Mixed R1 vs. MIxed P8 +ER p value=0.80; Mixed P8 vs. MIxed P8 +ER p value>0.99 | |
| Male | 426 ± 93 (n=6) | 601 ± 96 (n=6) | 567 ± 70 (n=6) |  |  |
| Female | 260 ± 54 (n=7) | 247 ± 64 (n=7) | 255 ± 116 (n=8) |  |  |
| Mixed-gender | 336.8 ± 58 (n=13) | 410.7 ± 77 (n=13) | 419.8 ± 77 (n=14) |  |  |
|  |  |  |  |  |  |
| **% of aNSC of total rGFAP^+^ Sox2^+^** | |  |  |  | |
| Male | 2.27 ± 0.48 (n=6) | 4.22 ± 0.43 (n=6) | 6.93 ± 0.84  (n=6) | ♂ R1 vs ♂ P8: p value=0.1 ♂ R1 vs ♂ P8+ER: p value=0.0002 ♂ P8 vs ♂ P8+ER: p value=0.02 ♀ R1 vs ♀ P8: p value=0.93 ♀ R1 vs ♀ P8+ER: p value=0.69 ♀ P8 vs ♀ P8+ER: p value=0.89 ♂ R1 vs ♀ R1: p value=0.99 ♂ P8 vs ♀ P8: p value=0.12 ♂ P8+ER vs ♀ P8+ER: p value <0.0001 Mixed R1 vs. Mixed P8 p value=044; Mixed R1 vs. MIxed P8 +ER p value=0.02; Mixed P8 vs. MIxed P8 +ER p value=0.26 | |
| Female | 1.72 ± 0.06 (n=6) | 2.03 ± 0.50 (n=7) | 2.39 ± 0.89  (n=8) |  |  |
| Mixed-gender | 2.02 ± 0.33 (n=12) | 3.3± 0.41 (n=13) | 4.6 ± 0.73 (n=14) |  |  |
| **DCX^+^ dendritic length (µm)** |  |  |  |  | |
| Male | 221.8 ± 19 (n=15) | 156.1 ± 19  (n=15) | 230.0 ± 14  (n=16) | ♂ R1 vs ♂ P8: p value =0.04 ♂ R1 vs ♂ P8+ER: p value=0.94 ♂ P8 vs ♂ P8+ER: p value=0.02 ♀ R1 vs ♀ P8: p value > 0.99 ♀ R1 vs ♀ P8+ER: p value > 0.99 ♀ P8 vs ♀ P8+ER: p value> 0.99 ♂ R1 vs ♀ R1: p value >0.99 ♂ P8 vs ♀ P8: p value>0.99 ♂ P8+ER vs ♀ P8+ER: p >0.99 Mixed R1 vs. Mixed P8 p value =0.43; Mixed R1 vs. MIxed P8 +ER p value=0.99; Mixed P8 vs. MIxed P8 +ER p value=0.1 | |
| Female | 189.23 ± 15 (n=19) | 193.5 ± 13 (n=12) | 196.5 ± 20 (n=14) |  |  |
| Mixed-gender | 204.1 ± 18 (n=34) | 172.7 ± 16(n=27) | 214,4 ± 17(n=31) |  |  |
| **DCX^+^ dendritic surface (µm2)** |  |  |  |  | |
| Male | 784.2 ± 63 (n=15) | 540.0 ± 63  (n=15) | 854.9 ± 36 (n=16) | ♂ R1 vs ♂ P8: p value =0.024 ♂ R1 vs ♂ P8+ER: p value=0.94 ♂ P8 vs ♂ P8+ER: p value=0.002 ♀ R1 vs ♀ P8: p value > 0.99 ♀ R1 vs ♀ P8+ER: p value > 0.99 ♀ P8 vs ♀ P8+ER: p value> 0.99 ♂ R1 vs ♀ R1: p value >0.99 ♂ P8 vs ♀ P8: p value>0.99 ♂ P8+ER vs ♀ P8+ER: p =0.32 Mixed R1 vs. Mixed P8 p value =0.14; Mixed R1 vs. MIxed P8 +ER p value=0.63; Mixed P8 vs. MIxed P8 +ER p value=0.003 | |
| Female | 662.9 ± 59(n=13) | 616.6 ± 36 (n=10) | 691.91 ± 54(n=15) |  |  |
| Mixed-gender | 723.9 68 (n=29) | 578.9± 50 (n=25) | 770.39± 47 (n=31) |  |  |
|  |  |  |  |  |  |
| **DCX^+^ segments** |  |  |  |  | |
| Male | 10.4± 1.0 (n=14) | 5.3 ±0.6 (n=14) | 8.3 ± 0.7 (n=15) | ♂ R1 vs ♂ P8: p value <0.001 ♂ R1 vs ♂ P8+ER: p value=0.16 ♂ P8 vs ♂ P8+ER: p value=0.04 ♀ R1 vs ♀ P8: p value > 0.99 ♀ R1 vs ♀ P8+ER: p value =0.07 ♀ P8 vs ♀ P8+ER: p value=0.427 ♂ R1 vs ♀ R1: p value >0.99 ♂ P8 vs ♀ P8: p value=0.002 ♂ P8+ER vs ♀ P8+ER: p >0.99 Mixed R1 vs. Mixed P8 p value =0.003; Mixed R1 vs. MIxed P8 +ER p value=0.03; Mixed P8 vs. MIxed P8 +ER p value>0.99 | |
| Female | 11.1 ± 0.9 (n=20) | 10.2 ± 0.9 (n=13) | 8.6 ± 0.6 (n=14) |  |  |
| Mixed-gender | 10.8± 0.6 (n=33) | 7,8 ± 0.7 (n=27) | 8,40 ± 0,5 (n=29) |  |  |
|  |  |  |  |  |  |
| **DCX^+^ terminal segments** | |  |  |  | |
| Male | 5.9 ± 0.5 (n=14) | 3.2 ± 0.3 (n=14) | 4.6 ± 0.3 (n=16) | ♂ R1 vs ♂ P8: p value <0.001 ♂ R1 vs ♂ P8+ER: p value=0.08 ♂ P8 vs ♂ P8+ER: p value=0.05 ♀ R1 vs ♀ P8: p value > 0.99 ♀ R1 vs ♀ P8+ER: p value =0.199 ♀ P8 vs ♀ P8+ER: p value=0.658 ♂ R1 vs ♀ R1: p value >0.99 ♂ P8 vs ♀ P8: p value=0.002 ♂ P8+ER vs ♀ P8+ER: p >0.99 Mixed R1 vs. Mixed P8 p value =0.004; Mixed R1 vs. MIxed P8 +ER p value=0.03; Mixed P8 vs. MIxed P8 +ER p value>0.99 | |
| Female | 6.0 ± 0.4(n=18) | 5.7 ± 0.5 (n=12) | 5.0± 0.3 (n=13) |  |  |
| Mixed-gender | 5.8 ± 0.4 (n=32) | 4.4 ± 0.3 (n=26) | 4.7 ± 0.2 (n=29) |  |  |
|  |  |  |  |  |  |
| **% DCX-BRDu respect total BRDU (%)** | |  |  |  | |
| Male | 45.5 ± 11 (n=12) | 35.2 ± 10 (n=11) | 48.8 ± 7 (n=12) | ♂ R1 vs ♂ P8: p value=0.74 ♂ R1 vs ♂ P8+ER: p value=0.98 ♂ P8 vs ♂ P8+ER: p value=0.63 ♀ R1 vs ♀ P8: p value=0.60 ♀ R1 vs ♀ P8+ER: p value =0.69 ♀ P8 vs ♀ P8+ER: p value=0.99 ♂ R1 vs ♀ R1: p value=0.34 ♂ P8 vs ♀ P8: p value>0=0.99 ♂ P8+ER vs ♀ P8+ER: p=0.67 Mixed R1 vs. Mixed P8 p value =0.97; Mixed R1 vs. MIxed P8 +ER p value=0.79; Mixed P8 vs. MIxed P8 +ER p value=0.68 | |
| Female | 20.1 ± 7(n=9) | 25.2 ± 6 (n=9) | 28.2 ± 6 (n=8) |  | |
| Mixed-gender | 34.6 ± 8 (n=21) | 30.7 ± 5(n=20) | 40.6 ± 5 (n=20) |  | |
| **% Neu-BRDu respect total BRDU (%)** | |  |  |  | |
| Male | 78.8 ± 6 (n=17) | 85.3 ± 3 (n=18) | 83.9 ± 3 (n=14) | ♂ R1 vs ♂ P8: p value>0.99 ♂ R1 vs ♂ P8+ER: p value>0.99 ♂ P8 vs ♂ P8+ER: p value>0.99 ♀ R1 vs ♀ P8: p value =0.40 ♀ R1 vs ♀ P8+ER: p value =0.87 ♀ P8 vs ♀ P8+ER: p value=0.25 ♂ R1 vs ♀ R1: p value>0.99 ♂ P8 vs ♀ P8: p value>0.99 ♂ P8+ER vs ♀ P8+ER: p=0.23 Mixed R1 vs. Mixed P8 p value =0.26; Mixed R1 vs. MIxed P8 +ER p value>0.99; Mixed P8 vs. MIxed P8 +ER p value=0.34 | |
| Female | 61.8 ± 8( n=12) | 80 ± 12 (n=5) | 55.8± 8 (n=8) |  | |
| Mixed-gender | 71.8 ± 5 (n=29) | 84.1± 4 (n=23) | 73.7 ± 5 (n=22) |  | |
|  |  |  |  |  | |
| **% S100-BRDu respect total BRDU (%)** | |  |  |  | |
| Male | 3.2 ± 2 (n=5) | 5.6 ± 2 (n=19) | 1.0 ± 0 (n=19) | ♂ R1 vs ♂ P8: p value>0.99 ♂ R1 vs ♂ P8+ER: p value=0.48 ♂ P8 vs ♂ P8+ER: p value=0.37 ♀ R1 vs ♀ P8: p value =0.83 ♀ R1 vs ♀ P8+ER: p value>0.99 ♀ P8 vs ♀ P8+ER: p value=0.65 ♂ R1 vs ♀ R1: p value >0.99 ♂ P8 vs ♀ P8: p value>0.99 ♂ P8+ER vs ♀ P8+ER: >0.99 Mixed R1 vs. Mixed P8 p value = 0.99; Mixed R1 vs. Mixed P8 +ER p value=099; Mixed P8 vs. MIxed P8 +ER p value= 0.14 | |
| Female | 1.6 ± 1 (n=15) | 3.5 ± 2 (n=15) | 1.0 ± 0 (n=15) |  | |
| Mixed-gender | 2.0 ± 1 (n=20) | 4.7 ± 1 (n=34) | 1.0 ± 0 (n=34) |  | |
|  |  |  |  |  | |
|  |  |  |  |  | |
